# Monitoring the evolution of individuals’ flood-related adaptive behaviors over time: two cross-sectional surveys conducted in the Province of Quebec, Canada

**DOI:** 10.1186/s12889-020-09763-6

**Published:** 2020-11-03

**Authors:** Pierre Valois, Maxime Tessier, David Bouchard, Denis Talbot, Alexandre J. S. Morin, François Anctil, Geneviève Cloutier

**Affiliations:** 1 Faculty of Education, Université Laval, 2320, rue des Bibliothèques, Quebec City, QC G1V 0A6 Canada; 2 Faculty of Medicine, Université Laval, 1050 Avenue de la Médecine, Quebec City, QC G1V 0A6 Canada; 3 Substantive-Methodological Synergy Research Laboratory, Department of psychology, Concordia University, 7141 Sherbrooke West, Montreal, QC H4B 1R6 Canada; 4 Water Research Centre, Department of Civil and Water Engineering, Université Laval, 1065 avenue de la Médecine, Quebec City, QC G1V 0A6 Canada; 5 Faculté d’aménagement, d’architecture, d’art et de design, Université Laval, Quebec City, QC G1V 0A6 Canada

**Keywords:** Adaptation, Climate change, Index, Validation, Flooding, Behavior

## Abstract

Climate change is predicted to increase the frequency and intensity of floods in the province of Quebec, Canada. Therefore, in 2015, to better monitor the level of adaptation to flooding of Quebec residents living in or near a flood-prone area, the Quebec Observatory of Adaptation to Climate Change developed five indices of adaptation to flooding, according to the chronology of events. The present study was conducted 4 years later and is a follow-up to the 2015 one. Two independent samples of 1951 (2015) and 974 (2019) individuals completed a questionnaire on their adoption (or non-adoption) of flood adaptation behaviors, their perception of the mental and physical impacts of flooding, and their knowledge of the fact that they lived in a flood-prone area.

The results of the study demonstrated the measurement invariance of the five indices across two different samples of people over time, ensuring that the differences (or absence of differences) observed in flood-related adaptive behaviors between 2015 and 2019 were real and not due to measurement errors. They also showed that, overall, Quebeckers’ flood-related adaptive behaviors have not changed considerably since 2015, with adaptation scores being similar in 2019 for four of the five flood indices. Moreover, the results indicated an increase in self-reported physical and mental health issues related to past flooding events, as well as a larger proportion of people having consulted a health professional because of these problems. Thus, this study provides a better understanding of flood adaptation in Quebec over the past 4 years and confirms that the five adaptive behavior indices developed in 2015 are appropriate tools for monitoring changes in flood adaptation in the province. Finally, our results showed that little has changed in Quebeckers’ adoption of adaptive behaviors, highlighting the need for awareness raising in order to limit the impacts that climate change will have on the population.

**Supplementary Information:**

**Supplementary information** accompanies this paper at 10.1186/s12889-020-09763-6.

## Background

Climate change will lead to a further increase in the number and intensity of catastrophic weather events such as heat waves, floods, and droughts [1–5]. Of these, flooding is already the most common and destructive of climate-related disaster in many countries, including Canada. For instance, 80% of waterfront municipalities in the Province of Quebec (Canada) are exposed to flooding [6]. Over the course of the twentieth century, more than 2 billion $CAD in damages have been caused by floods in this province, with the Saguenay River flood of 1996 alone accounting for around 1 billion $CAD [7]. In 2017, floods were estimated to have caused more than 376 million $CAD in damages to municipalities located in southern Quebec [8]. In the spring 2019, flooding occurred in more than 250 Quebec municipalities [9], largely the same as in 2017.

Flooding is also linked to several health issues. It may cause physical wounds, traumas or drowning, as well as mold-induced respiratory problems, gastrointestinal diseases, leptospirosis, skin infections, carbon monoxide poisoning, and electrocutions [10–16]. In addition, research indicates that flooding can lead to mental health issues such as post-traumatic stress disorder (PTSD), anxiety, and depression [17–19]. Between 1980 and 2009, nearly 2.8 billion people were affected by floods worldwide, and 539,811 people lost their lives [20]. While this number includes extremely deadly events such as the flood following cyclone Nargis in Myanmar in 2008 (≈ 100,000 deaths), Canada has been relatively successful in preventing floods from directly causing such a high number of deaths, even though Canadians remain susceptible to the indirect and post-event health effects of flooding. In a review of the health impacts of the 2005 flood in southern Alberta, Acharya et al. [21] found that 63% of participants reported flood-related mental health effects. This is consistent with data from Hajat et al. [22] and Azuma et al. [23], who found that PTSD rates were significantly higher in populations having experienced a flood than in the general population. In Quebec, the Saguenay River floods of 1996 led to 10 deaths and 15,825 evacuations [24], while the Richelieu River floods of 2011 forced 11 municipalities to declare a state of emergency: 2524 primary residences were flooded, 3927 persons were affected, 1651 persons were evacuated, and 7000 psychosocial interventions took place [25]. More recently, the spring floods that occurred in 2019 in the Province of Quebec and in the Province of New Brunswick, its eastern Canadian neighbor, damaged 460 km of roads and affected about 17,500 homes. Importantly, more than 25% of the homes in the most affected municipalities were damaged [26].

The Quebec Observatory of Adaptation to Climate Change (OQACC; Observatoire québécois de l’adaptation aux changements climatiques) was established by the National Institute of Public Health of Quebec and the Green Fund as part of the Quebec government’s 2013–2020 Climate Change Action Plan. The OQACC’s mission is to improve knowledge of climate change adaptation practices in Quebec. From a public health and health promotion perspective, it is essential to better document to what extent individuals, organizations, and municipalities are prepared to face the consequences of climatic hazards such as heat waves, floods, vector-borne diseases like Lyme disease and pollen allergies.

In 2015, the OQACC monitored the temporal evolution of the level of adaptation to floods of the Quebec population living in or near a flood-prone area. In a first study [27], five indices of adaptation to flooding were developed based on literature from across the world. These indices echo recommended behaviors that any individual should adopt according to the chronology of events: (a) pre-alert preventive behaviors, (b) post-alert behaviors, (c) behaviors during a flood not requiring evacuation, (d) behaviors during a flood requiring evacuation, and (e) post-flood behaviors. Results revealed that 115 of the 797 (14.45%) people who had experienced at least one flood in their current home reported that their physical health had been moderately to greatly affected by the event, while 138 of them (17.32%) reported that their mental health had been moderately to greatly affected by the flood. Among those who reported that their physical health was affected, 50 (43.51%) had consulted a health professional about it (e.g., doctor, physiotherapist, chiropractor) while 35 (25.51%) had consulted a professional (e.g., doctor, psychologist) after their mental health being affected. Results also showed that 26.34% of the respondents who lived in an at-risk area were unaware of that fact.

The purpose of the present follow-up study, conducted 4 years later, was threefold. First, we aimed to test the measurement invariance of the five indices for different samples of people over time to ensure that the differences observed in flood-related adaptive behaviors between 2015 and 2019 were real and not due to measurement errors. Importantly, by doing so, the present study also contributes to establishing the psychometric properties of these indices and, thus, to make them available for use for research conducted in other regions and countries. Second, we aimed to estimate the change in the prevalence of health problems for people having experienced a flood. Our third objective was to investigate whether the proportion of the population unaware of living in a risk-prone area was lower in 2019 than in 2015, since a major flood occurred in 2017 and again in 2019.

## Methods

### Study design

To monitor the evolution of individuals’ flood-related adaptive behaviors over time in the Province of Quebec (1,667,441 km^2^; population of 8.3 million), we conducted two cross-sectional province-wide surveys, the first in 2015 and the second in 2019. Participants were selected from all 136,505 (2015) and 136,476 (2019) households whose main residence is found within or near a designated flood-prone area, as per the Quebec Government Water Agency (Ministère de l’Environnement et de la Lutte contre les changements climatiques).

### Samples and data collection

In 2015 and 2019, we used a stratified sample to preserve the geographical distribution of the flood-prone zones throughout the province. The samples consisted of 1951 (2015) and 974 (2019) individuals who were surveyed by a polling firm: 1450 (2015) and 724 (2019) lived in an at-risk area (flood recurrence: every 20 to 100 years), and 501 (2015) and 250 (2019) lived less than 150 m from a designated flood-prone area. Participants of the 2015 survey were not eligible for the 2019 survey. To avoid repeat participation, the phone numbers of the 2015 participants were excluded from the sampling frame.

Of the surveyed population in 2015, 83.85% (*n* = 1636) of the respondents were homeowners (80.80% in 2019; *n* = 787) and 16.15% (*n* = 315) were renters (19.20% in 2019; *n* = 187). Lastly, in 2015, 2.90% of the respondents were newcomers (had lived in their home for a year or less) compared to 5.66% in 2019. See Valois el al [27]. for a more detailed description of the data collection strategy.

To reduce costs in 2019, we used a sample that was half the size of the one used in 2015 because the estimated level of accuracy of the item responses did not differ considerably: 0.16 versus 0.12 according to Cochran’s formula [28], with a 99% level of confidence and a maximal variance for a 5-point scale.

The interviews lasted an average of 21 min in 2015 and 22 min in 2019. The response rate was 21.82% in 2015 and 24.5% in 2019. The interviews used the same questionnaire as that of the 2015 study [27]. The questionnaire is available as a supplementary file.

### Measures

#### Five flood adaptation indices

The five flood adaptation indices are valid constructs designed to assess the degree to which people living in an at-risk area adopt recommended behaviors according to the chronology of events. Fifteen are pre-alert actions (e.g., waterproof the foundations) or expertise (e.g., knowing how to cut off the main water valve), if the need arises. Nine are behaviors to adopt as soon as a flood alert is given (e.g., waterproofing the doors and windows with plastic tape). Five are behaviors suited for floods when there is no requirement for an evacuation (e.g., wearing rubber boots to walk in the flood water). Four are suited in a flood requiring evacuation (e.g., registering with a temporary shelter if available). Finally, ten are recommended after a flooding event (e.g., checking if mold has developed). The behaviors composing the five indices are listed in Tables 1, 2, 3, 4 and 5 below. All of them are scored using a binary (present-absent) response scale. We employed standards of validity that have been used by many researchers across various sectors (health, psychology, sociology, marketing, education, etc.) to construct these indices [29–32].

**Table 1:** List of pre-flood preventive behaviors

1. Make a list of your belongings that could be used for a claim in case of flooding	
2. Make a plan for evacuating your neighborhood in case of emergency	
3. Know how to cut off the electricity or the water	
4. Inquire about how to better prepare for a flood or to make your home more flood-resistant	
5. Inquire about the consequences that a flood could have on your physical or mental health	
6. Waterproof the foundations	
7. Raise the baseboard heaters or electrical outlets on the walls	
8. Replace water-sensitive flooring	
9. Install a backwater valve	
10. Relocate the home elsewhere on the property	
11. Make other changes to the building	
12. Change the landscape to help water runoff	
13. Check to be sure the foundation drain is not blocked	
14. Make other changes to the property to make it more flood-resistant	
15. Own a water pump

**Table 2:** List of behaviors to perform at the time of a flood alert

1. Move your lawn or patio furniture or your vehicle to higher ground	
2. Store items or furniture higher or on a higher floor	
3. Block the basement drain	
4. Cut off the electricity if requested by the authorities	
5. Waterproof the doors and windows with plastic tape	
6. Block the outside air inlets like the one for the clothes dryer, the range hood, the air exchanger, etc.	
7. Put sandbags on the property or help your neighbors implement their protective measures	
8. Implement other measures to prevent the water from entering (e.g., board up the windows, prepare the water pump, etc.)	
9. Check regularly if the risk of flooding has increased or decreased

**Table 3:** List of behaviors to carry out during a flood not requiring an evacuation

1. Boil the water or use bottled water	
2. Wear rubber gloves to handle items in contact with the flood water	
3. Wear rubber boots to walk in the flood water	
4. Install a pump to drain the water from the home

**Table 4:** List of behaviors to carry out when evacuating one’s home

1. Bring your emergency kit, including your medication	
2. Lock the doors	
3. Tell your loved ones where you can easily be reached	
4. Use the route indicated by the authorities to evacuate the neighborhood	
5. Wait for the authorities’ permission before returning home

**Table 5:** List of post-flood behaviors

1. Have the condition of the electrical installation and heating appliances checked	
2. Replace the refrigerator insulation if it is wet or replace the appliance	
3. Disinfect the contaminated rooms	
4. Sterilize all kitchen items contaminated by the flood water	
5. Discard items in contact with the flood water	
6. Wear rubber gloves to handle items in contact with the flood water	
7. Check if mold has developed	
8. Make a list of the damages caused to the home and to your belongings	
9. Update your emergency kit	
10. Attend citizens’ meetings concerning the flood	

Respondents had to answer questions that concerned them directly, as filter questions were used throughout the questionnaire (for example, some participants had never been evacuated). Filters were also used in the questionnaire so that renters were asked if they knew whether their landlord had adopted the behavior or not, while some questions were only posed to homeowners, such as whether they owned a water pump to evacuate flood water and whether they blocked the basement water drain when they received the flood alert. Items making up these indices were identified following a review of the literature on flooding adaptation (e.g. [33–36]) and government guidelines [37–40]. For more details on how these flood adaptation indices were constructed and validated, see Valois et al. [27].

#### Knowing that they live in an at-risk area

The respondents who lived in an at-risk area (flood recurrence: on average every 20 to 100 years) were asked: “To your knowledge, do you live in a flood-risk zone?” They had three response options to answer this question: yes, no, don’t know.

#### Physical and mental health problems

The respondents who reported having been previously flooded were asked the following two questions, evaluated on a 4-point response scale (1 = not at all, 2 = slightly, 3 = moderately, 4 = very much): “Was your physical health negatively affected by the flood?” and “Was your mental health negatively affected by the flood?”. After answering these two questions, the participants were asked to indicate the nature of these physical (or mental) health problems. Finally, respondents indicated in a Yes-No response format whether they had consulted a health professional regarding one of these physical (or mental) health problems.

### Statistical analyses

We tested the measurement invariance (equivalence) of the five flood adaptation indices across the 2015 and 2019 independent samples of participants who completed the questionnaire to ensure that the differences observed in flood-related adaptive behaviors between 2015 and 2019 were real and not due to measurement errors. According to Morin et al. [41] and Millsap [42], measurement invariance is a necessary condition to ensure unambiguous interpretation of the differences (or lack thereof) observed in flood-related adaptive behaviors.

These tests were carried out in the following order. First, a model with no parameter invariance, also called a configural invariance model, was estimated. Typically, two measurement invariance tests are used to estimate the weak invariance (factor loadings) and the strong invariance (factor loadings and response thresholds) but separating these steps is not possible when binary items are used [43]. Given the binary nature of the items, in a second step we thus simultaneously tested the strong invariance of the factor loadings and response threshold (i.e., the weak-strong invariance model) [43]. Non-respect of factor loading invariance suggests that the index does not assess the same constructs across groups. Non-respect of response threshold invariance suggest that participants’ item response process differed systematically across groups irrespective of their true score on the underlying construct (i.e., an item bias). Both forms of invariance are required for valid group comparisons, although partial invariance of a majority of factor loadings and of response threshold is sufficient to ensure valid comparisons [44].

Third, we tested the strict invariance of the items’ uniquenesses (i.e., residuals). Non invariance of items’ uniquenesses suggests that the measurement errors present in the item responses are non-equivalent across groups.

Fourth and fifth, we tested the invariance of the latent variance and of the latent means across groups. The purpose of these tests was not to assess the presence of measurement bias (as in the first steps), but to assess the presence of meaningful group-based differences in within-group variability and means. For each step of the invariance testing process, each model was compared with the model from the previous step. We used the method suggested by Little et al. [45] to compare the latent means between the 2015 and 2019 samples. According to Litalien et al. [46] “This method allows estimating latent means in a nonarbitrary metric that reflects the metric of the indicators measured”. In addition, comparison of latent means can generate more accurate results than comparison of composite scores using a t-test or analysis of variance (ANOVA), as the latent variables are free of measurement errors [47].

All models were estimated using the robust weighted least squares estimator with the mean and variance adjusted statistics (WLSMV) implemented in the Mplus 8.4 statistical package [48, 49]. Since chi-square is known to be oversensitive to sample size and minor model misspecifications, model fit was assessed using sample-size-independent fit indices: the comparative fit index (CFI), the Tucker-Lewis index (TLI), and the root mean squared error of approximation (RMSEA). CFI and TLI index values greater than or equal to 0.90 and less than 0.95 indicate an acceptable model fit. Values greater than 0.95 for these indices indicate an excellent model fit. As for RMSEA, the model fit is acceptable when values are between 0.05 and 0.08 and is excellent when values are less than or equal to 0.05 [48, 50]. For each step of the invariance process, invariance is respected if both the CFI and TLI indexes do not decrease by 0.010 or more and the RMSEA index does not increase by 0.015 or more. Indices score reliability were computed using McDonald’s omega (ω) [51]. This coefficient estimates the reliability of a questionnaire (e.g., an attitude scale) or an index. It is used to verify that the observed differences between individuals are due to differences in true scores and not to measurement errors. A value above 0.70 is considered acceptable [52].

We finally performed proportion comparison z-tests and effect size tests to evaluate whether there are differences between proportions of people in 2015 and 2019 who knew that they lived in a flood-prone zone, reported adverse health effects of flooding, and reported having consulted a health professional after being flooded.

## Results

The two samples were very similar in terms of sociodemographic characteristics. All respondents were aged 18 years or older. The mean age of the participants in the 2015 and 2019 samples was respectively 57.3 and 60.7 years. Men made up 44.54% of the sample in 2015 and 40.66% in 2019. In 2015, 30.09% of the respondents reported an annual net income of 40,000 $CAD or less, 29.21% an income between 40,001 and 80,000 $CAD, and 26.71% an income greater than 80,000 $CAD, compared with 27.9, 28.6, and 24.9%, respectively, in 2019. All these differences were statistically significant but negligible according to effect size analyses: Cramer’s V = 0.04 for gender and 0.06 for income; Cohen’s d = 0.24 for age. Finally, no statistically significant differences were found between the 2015 and 2019 samples in regard to the percentage of participants for whom the highest education level obtained was a university degree: 31.37% versus 28.75%.

### Measurement invariance

We tested the measurement invariance of the five indices of adaptation to flooding across the independent samples of participants who completed the survey measuring their flood-related adaptive behaviors in 2015 and 2019. Results from these tests are reported in Tables 6, 7, 8, 9 and 10.

**Table 6:** Goodness-of-fit statistics for the models of the pre-flood preventive behaviors to adopt

Models	χ^2^	df	RMSEA	CFI	TLI	ΔRMSEA	ΔCFI	ΔTLI	Compared Model
Single-group models									
2015 sample	288.900	90	0.034	0.933	0.921				
2019 sample	192.495	90	0.034	0.926	0.914				
Multiple-group measurement models									
Configural invariance	432.133	181	0.031	0.940	0.930	–	–	–	–
Weak-strong invariance	446.603	194	0.030	0.940	0.935	−0.001	0	0.005	1
Strict invariance	467.042	209	0.029	0.938	0.938	−0.001	− 0.002	0.003	2
Latent variance invariance	453.711	210	0.028	0.942	0.942	−0.001	0.004	0.004	3
Latent mean invariance	449.158	211	0.028	0.943	0.943	0	0.001	0.001	4

**Table 7:** Goodness-of-fit statistics for the models of the behaviors to perform at the time of a flood alert

Model	χ^2^	df	RMSEA	CFI	TLI	ΔRMSEA	ΔCFI	ΔTLI	Compared Model
Single-group models									
2015 sample	46.17	22	0.035	0.932	0.909				
2019 sample	38.85	22	0.037	0.956	0.942				
Multiple-group measurement models									
Configural invariance	86.728	54	0.036	0.944	0.925	–	–	–	–
Weak-strong invariance	119.909	61	0.045	0.899	0.881	0.009	−0.045	−0.044	1
Partial weak-strong invariance	90.807	58	0.035	0.944	0.930	−0.001	0.000	0.005	1
Strict invariance	105.099	67	0.035	0.935	0.930	0.000	−0.009	0.000	3
Latent variance invariance	108.505	68	0.036	0.930	0.926	0.001	−0.005	−0.004	4
Latent mean invariance	111.287	69	0.036	0.927	0.924	0.000	−0.003	−0.002	5

**Table 8:** Goodness-of-fit statistics for the models of the behaviors to perform at the time of a flood not requiring an evacuation

Models	χ^2^	df	RMSEA	CFI	TLI	ΔRMSEA	ΔCFI	ΔTLI	Compared Model
Single-group models									
2015 sample	1.95	5	0.052	0.976	0.928				
2019 sample	3.33	5	0.112	0.949	0.847				
Multiple-group measurement models									
Configural invariance	13.172	4	0.072	0.967	0.902	–	–	–	–
Weak-strong invariance	18.896	6	0.070	0.954	0.908	−0.002	−0.013	0.006	1
Partial weak-strong invariance	14.504	5	0.066	0.966	0.919	−0.006	−0.001	0.017	1
Strict invariance	20.673	9	0.054	0.958	0.945	−0.012	−0.008	0.026	3
Latent variance invariance	20.404	10	0.049	0.963	0.956	−0.005	0.005	0.011	4
Latent mean invariance	19.584	11	0.042	0.969	0.967	−0.007	0.006	0.011	5

**Table 9:** Goodness-of-fit statistics for the models of the behaviors to perform at the time of a flood requiring an evacuation

Models	χ^2^	df	RMSEA	CFI	TLI	ΔRMSEA	ΔCFI	ΔTLI	Compared Model
Single-group models									
2015 sample	5.18	2	0.000	1.000	1.000				
2019 sample	9.46	2	0.000	1.000	1.000				
Multiple-group measurement models									
Configural invariance	5.544	10	0.000	1.000	1.000	–	–	–	–
Weak-strong invariance	7.318	13	0.000	1.000	1.000	0	0	0.000	1
Strict invariance	8.777	18	0.000	1.000	1.000	0	0	0.000	2
Latent variance invariance	9.209	19	0.000	1.000	1.000	0	0	0.000	3
Latent mean invariance	12.179	20	0.000	1.000	1.000	0	0	0.000	4

**Table 10:** Goodness-of-fit statistics for the models of the behaviors to perform after the flood

Models	χ^2^	df	RMSEA	CFI	TLI	ΔRMSEA	ΔCFI	ΔTLI	Compared Model
Single-group models									
2015 sample	44.450	35	0.026	0.991	0.988				
2019 sample	44.817	35	0.040	0.982	0.977				
Multiple-group measurement models									
Configural invariance	125,657	73	0.050	0.966	0.959	–	–	–	–
Weak-strong invariance	121,576	81	0.042	0.974	0.971	−0.008	0.008	0.012	1
Strict invariance	136,668	91	0.042	0.971	0.971	0	−0.003	0	2
Latent variance invariance	132,845	92	0.040	0.974	0.975	−0.002	0.003	0.004	3
Latent mean invariance	160,498	93	0.051	0.957	0.958	0.011	−0.017	−0.017	4

For the pre-flood preventive behavior index, results showed that throughout the full sequence of invariance tests, no ΔCFI or ΔTLI exceeded − 0.010 and no ΔRMSEA exceeded + 0.015 (see Table 6). Thus, the full invariance of this index was respected over time. The invariance of the variance-covariance and the latent means indicated that there were no meaningful group-based differences in terms of within-group variance and mean.

Moreover, according to an ANOVA-like latent mean comparison across groups of participants within a latent variable framework [45, 46], which is expressed as a between-group deviation in standard deviation units, the participants surveyed in 2019 and those surveyed in 2015 reported, on average, the same levels of adoption of pre-flood preventive behaviors (deviation = − 0.01, *p* = 0.81). This conclusion is consistent with the previously reported result supporting the invariance of the latent means.

**Fig. 1:**
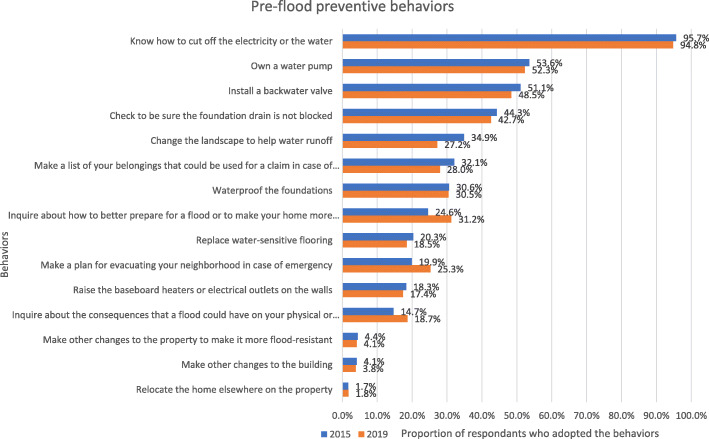
Proportion of respondents who adopted the recommended pre-alert preventive behaviors

The means of each pre-flood preventive behavior in 2015 and 2019 are reported in Fig. 1. As expected, given the previous result of latent mean invariance, results showed that the percentage of people adopting each of these behaviors in 2015 and in 2019 were very similar. The results also showed that few participants (i.e., between 2 and 54%) reported adopting most of the pre-flood preventive behaviors, for instance, raise the baseboard heaters or electrical outlets on the walls (≈18%), waterproof the foundations (≈30%), change the landscape to help water runoff, check to be sure the foundation drain is not blocked (≈43%). The only exception was knowing how to cut off the electricity or the water, which 95% of the participants reported adopting. The pre-flood index presented a good reliability (ω = 0.829; above the .70 threshold).

The index of adaptation at the time of the alert did not support strong invariance according to a ΔCFI of − 0.045 and a ΔTLI of − 0.044. This implies that either one or many estimated factor loadings or response thresholds differed between 2015 and 2019. A detailed examination of the model parameters and model modification indices suggested that invariance constraints needed to be relaxed only for the response threshold associated with three items: (a) cut off the electricity if requested by the authorities, (b) put sandbags on the property, and (c) check regularly if the risk of flooding has increased or decreased. We therefore re-estimated a partial invariance model in which the response threshold associated with these three items was allowed to be freely estimated over time. This model supported the partial weak-strong invariance of this index. Starting from this model, the strict invariance of the items’ uniquenesses, as well as the invariance of the within group variance and latent means, was also supported by the data.

Moreover, results of the ANOVA-like latent mean comparison across the 2015 and 2019 samples indicated that, on average, the participants surveyed in 2015 and those surveyed in 2019 reported similar levels of behavior adoption at the time of the alert (deviation = 0.15, *p* = 0.14).

**Fig. 2:**
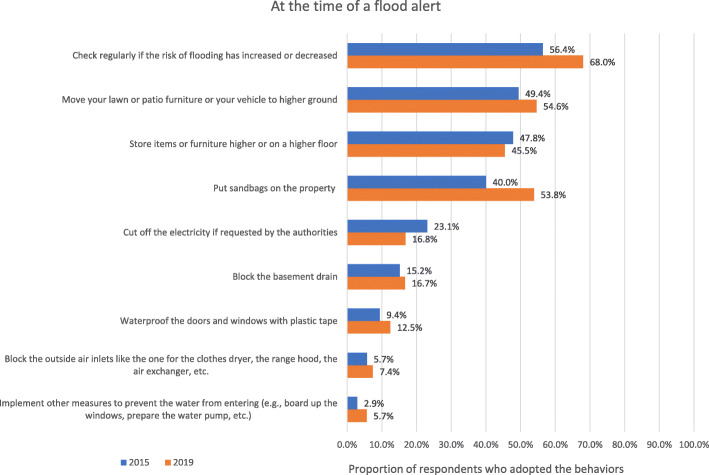
Proportion of respondents who adopted the recommended behaviors at the time of the alert

The means of each behavior to perform at the time of a flood alert reported in 2015 and 2019 are indicated in Fig. 2. In agreement with the results supporting the latent mean invariance, results showed that the percentage of people adopting each of these behaviors in 2015 and in 2019 was very similar. An examination of the means revealed low proportions of respondents having adopted the recommended behaviors during an alert (i.e., less than 50% for 8 out of the 10 behaviors). The index of adaptation at the time of the alert presented a good reliability (ω = 0.769).

**Fig. 3:**
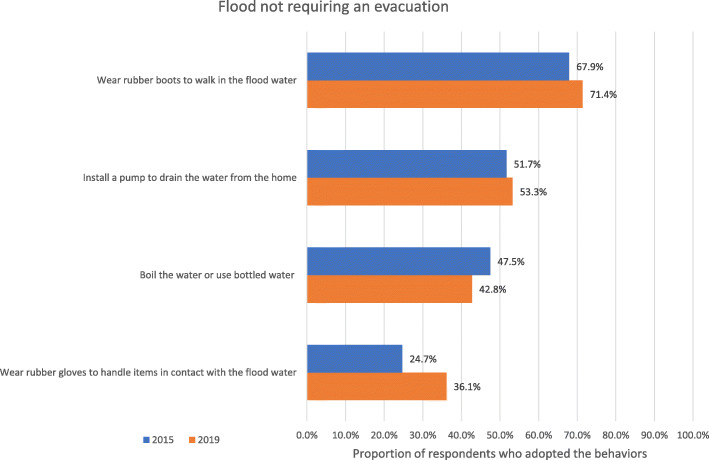
Proportion of respondents who adopted the recommended behaviors during a flood not requiring evacuation

Results for the index of adaptation during a flood not requiring an evacuation are reported in Table 8 (see Fig. 3 for the means of each behaviors). When weak-strong measurement invariance constraints were included in the model, the decrease in fit exceeded very slightly the recommended cut-off for the CFI (ΔCFI = − 0.013), indicating non-invariance of factor loadings or response thresholds between 2015 and 2019. A detailed examination of the model parameters and model modification indices suggested that invariance constraints needed to be relaxed only for the response threshold associated with a single item (i.e., Wearing rubber gloves), The resulting model of partial weak/strong was supported by the data. Starting from this model, the strict invariance of the items’ uniquenesses, as well as the invariance of the within group variance and latent means, was also supported by the data.

Moreover, results of the ANOVA-like latent mean comparison across the 2015 and 2019 samples indicated that, on average, the participants surveyed in 2019 and those surveyed in 2015 reported similar levels of behavior adoption during a flood not requiring an evacuation (deviation = 0.18, *p* = 0.09). This indicates that the proportion of participants that had adopted the behaviors recommended for this index was similar in 2019 and in 2015. The proportion of adoption of these behaviors was relatively low, varying between 25 and 68% in 2015 and between 36 and 71% in 2019. The index of adaptation during a flood not requiring an evacuation presented a good reliability (ω = 0.709).

**Fig. 4:**
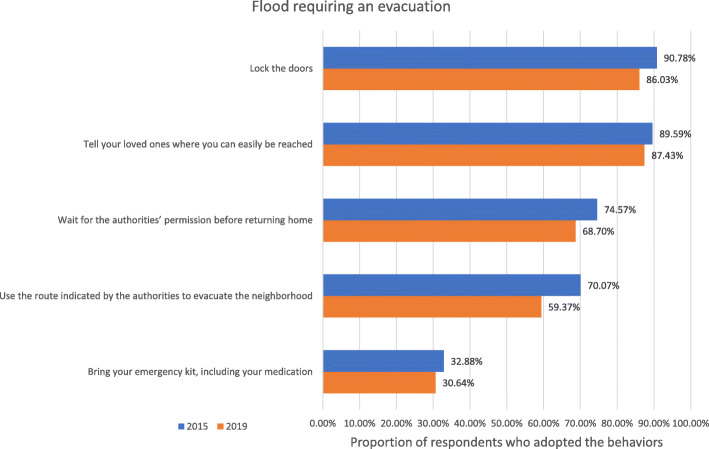
Proportion of respondents who adopted the recommended behaviors during a flood requiring evacuation

Finally, we performed invariance tests across time for the index of adaptation during a flood requiring evacuation as well as the index of post-flood adaptation. Results from these analyses supported the full invariance of the index of adaptation during a flood requiring evacuation and the weak, strong, strict, and latent variance invariance of the index of post-flood adaptation, but not its latent mean invariance (see Tables 9 and 10 and Fig. 4).

A notable decrease in the levels of behavior adoption during a flood requiring an evacuation was observed between 2015 and 2019 in the ANOVA-like latent mean comparison, but was not statistically significant (deviation = − 0.42, *p* = 0.09). Conversely, in accordance with the lack of latent mean invariance, the results showed that the participants surveyed in 2019 reported higher levels of adoption of post-flood behaviors than the participants surveyed in 2015 (deviation = 0.34, *p* = 0.002).

The proportion of participants who adopted the recommended behaviors during a flood requiring an evacuation varied between 60 and 90% for four out of five behaviors: lock the doors, tell your loved ones where you can be easily reached, wait for the authorities’ permission before returning home, and use the route indicated by the authorities to evacuate the neighborhood. However, a small percentage of them (i.e., around 30%) reported bringing their emergency kit, including their medication, during the evacuation.

**Fig. 5:**
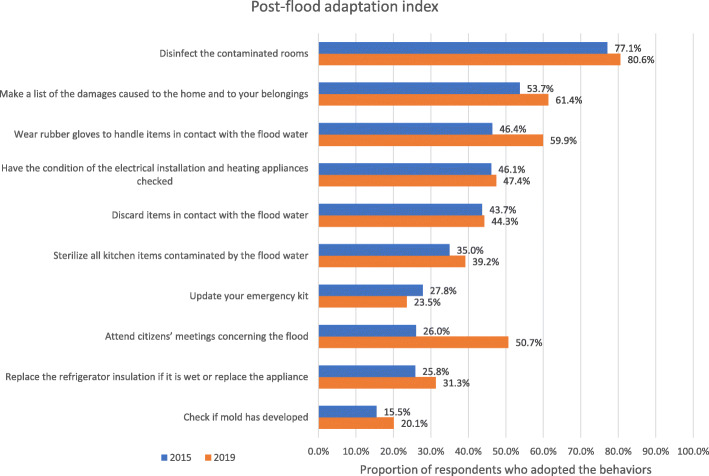
Proportion of respondents who adopted the recommended post-flood behaviors

For nine of the 10 post-flood behaviors, the proportion of respondents adopting them was quite low: between 16 and 54% in 2015 and between 20 and 61% in 2019 (see Fig. 5 for the means of each behaviors). The least adopted (by less than 35% of the participants) were: sterilize all kitchen items contaminated by the flood water, update your emergency kit, attend citizens’ meetings concerning the flood, replace the refrigerator insulation if it is wet or replace the appliance, and check if mold has developed. The index of adaptation during a flood requiring an evacuation presented an acceptable reliability (ω = 0.679; slightly below the 70 threshold). The post-flood index also presented a good reliability (ω = 0.848). Detailed results for each index regarding the standardized loadings and uniquenesses are available in the Supplemental Materials.

In sum, even though the Quebec media extensively covered the 2017 and 2019 floods over many weeks, the rates of adaptive behavior did not substantially change. Thus, as a measure of control, we performed analyses of covariance (ANCOVAs) to verify if this non-difference in adaptive behavior between 2015 and 2019 (for four of the five indices) was due or not to the effect of the respondents’ property status (i.e., more newcomers in 2019 than in 2015). Results from the ANCOVAs showed that there was no significant difference between the adaptive behaviors of the 2015 and 2019 samples after controlling for property status, except for the adaptive behaviors related to the post-flood index, which supports our latent means results (all *p*-values > .05 except for the post-flood index): F(1, 2037) = 0.14 for the pre-flood index; F(1, 537) = 3.85 for the alert index; F(1, 719) = 2.58 for the flooding with evacuation index; F(1, 179) = 1.51for the flooding without evacuation index; F(1, 485) = 10.54 for the post-flood index.

### Knowing that they lived in an at-risk area

In 2015 and 2019, the proportions of respondents who knew that they lived in a flood-prone area were 24.34 and 34.76%, respectively. Results from the z-test showed that this difference was statistically significant (z = − 3.49, *p* < 0.0001) but negligible according to the effect size analysis (Cohen’s h = 0.18).

We also performed an analysis of covariance to verify that the higher percentage of people aware that they live in flood-prone zones in 2019 as compared to 2015 was not due to the effect of the respondents’ property status. The ANCOVA results showed that there was still a significant difference after controlling for property status: F(1, 2143) = 10.66, *p* = .001.

### Prevalence of health problems

The prevalence of physical problems reported by the participants having been previously flooded was 14.67% in the 2015 survey and 24.95% in the 2019 survey (i.e., the proportion of respondents who reported that their physical health was moderately or very much affected by the flood on a 4-point Likert scale). Results showed also that the prevalence of the mental health and well-being problems reported by these participants was 17.32% in 2015 and 31.69% in 2019. Results from the z-test showed that these differences were statistically significant (physical problem: z = − 3.90, *p* < 0.0001; mental health problem: z = − 4.92, *p* < 0.0001), but small according to the effect size analysis (Cohen’s h = 0.26 and 0.34).

Among the respondents who reported physical problems, 44.18% reported consulting a health professional in 2015 compared with 48.65% in 2019. Among the respondents who reported mental health problems, these percentages were 25.51 and 35.40%. Results from the z-tests showed that these differences were not statistically significant (physical problem: z = − 0.48, *p* = 0.317; mental health problem: z = − 1.44, *p* = 0.075), but there was a small effect size for the mental health problems (Cohen’s h = 0.09 and 0.22).

## Discussion

When we created the five flood adaptation indices in 2015, there was, to our knowledge, no surveillance of citizens’ flood adaptation behaviors in Quebec. Because flooding is the most common and destructive natural disaster in Canada [7], it was important to create a valid tool to better monitor how well the at-risk population adopt measures promoted by authorities. This was achieved in 2015, but at that time we could not verify the measurement invariance of the indices over time, since making such an analysis requires two measurements over time. In fact, unless indices measure the same construct and operate in the same manner over time for different samples, they may not be valid for monitoring the evolution of individuals’ behaviors over time at the population level. Our results demonstrated the measurement invariance of each of the five indices across two samples of participants from Quebec, Canada. This means that the results obtained from our follow-up study are not due to measurement error and can be reliably compared over time. Thus, it appears that all five adaptive behavior indices are appropriate for the culture and climate of Quebec, making them suitable tools for monitoring changes in flood adaptation in the province. A next step would be to verify the extent to which these desirable psychometric properties generalize to other regions, province, or countries.

Whereas monitoring will require the same survey to be conducted more than twice over time, we can already conclude that Quebeckers’ flood adaptation behaviors did not change substantially between 2015 and 2019. Indeed, our results indicated that, overall, they scored similarly in 2015 and 2019 for four out of the five flood adaptation indices. While some small changes between specific behaviors from each index were observed, a comparison of the adaptation scores showed that adaptation had not improved considerably in those 4 years. Adoption of post-flooding adaptive behaviors had changed noticeably since 2015, with most of the behaviors in the post-flood adaptation index being adopted more frequently. For instance, there had been a large increase, of 24.7%, in the frequency of people attending citizens’ meetings concerning the flood they had experienced.

Though no mass awareness campaign had been conducted during the period of 2015–2020, the spring floods of 2017 and 2019 were widely covered by Quebec media and affected thousands of homes [9, 26, 53–55]. Considering that the second survey took place after these widely mediatized events, we could have expected them to influence the rates of adaptive behavior adoption by enhancing Quebeckers’ knowledge of flood risks. Yet, adaptation rates remained similar for most indices. Moreover, despite the widely publicized floods of 2017 and 2019 [9, 53], the percentage of at-risk population who did not know they lived in a flood-prone area had increased since 2015. Indeed, whereas 26.3% of the at-risk population did not know that they lived in a flood-prone area in 2015, 34.8% did not know it in 2020. While this difference is negligible (i.e., small effect size), it highlights a growing issue specific to the context of flood maps in the Province of Quebec, which are often outdated, difficult to access and hard to understand for nonexperts [56–58]. In fact, these results show that it is imperative that citizens be better informed of the existence of interactive maps identifying areas at risk and also that guides be elaborated by local authorities to help the general population use these maps, as they are not that trivial to interpret. Results also show that from a flood awareness education perspective, a better understanding of the determinants that favor or limit the adoption of preventive behaviors by citizens (e.g., their beliefs about the likely positive and negative consequences of performing the behaviors, and their perception of the presence of factors that facilitate or impede adoption of the behaviors) is essential. As stated by Ajzen et al. [59], changing people’s lifestyles and behavioral patterns requires more than simply transmitting information. It is thus essential for future research to target the source of decision-making and to identify the specific beliefs that shape the adoption of adaptive behaviors.

Results presented here are in line with findings showing that floods have a major impact on psychological and physical well-being [11, 21, 23, 60–62]. A longitudinal study in England showed high psychological morbidity in groups affected by flooding, even 2 to 3 years after exposure, particularly when it comes to depression and anxiety [63–65]. While our study design did not have a control group with which to compare, respondents who had experienced a flood reported high rates of physical and mental health issues. In fact, there had been a noticeable increase in reported health issues since 2015, as self-reported physical or mental health impacts from flooding rose from 22.9% in 2015 to 40.7% in 2020. These issues ranged from depression, PTSD, anxiety, and sleep difficulties, to exhaustion, headaches, body pain, illnesses, or respiratory and heart problems. Moreover, this increase in reported health issues was accompanied by an increase in the proportion of people having consulted a health professional (e.g., doctor, psychologist) because of flood-related mental or physical health problems: 50.7% of respondents reported consulting a health professional in 2015, versus 59.2% in 2020. The increase in reported health impacts of flooding and consultation of health professionals since 2015 is hard to explain and may be due to differences in the severity of the floods experienced by respondents in the 2020 survey, as health impacts are highly specific to particular contexts. Still, while short-term health impacts of flooding are well understood [11, 13], long-term health effects, such as PTSD, depression, and anxiety, are not as well documented. Longitudinal data from England support the need to provide suitable treatment to flood victims in order to prevent chronic health issues from developing, because even over a three-year follow-up period, there was persistence of psychological morbidity [65]. While nearly half (48.7%) of the respondents in the present study reporting physical health impacts from flooding consulted a health professional, only 35.4% of those reporting mental health impacts did so. Both numbers had increased since 2015, but the discrepancy between them may indicate a need to improve the availability of psychological help in order to prevent chronic mental health issues from developing in this population.

This study presents several limitations that need to be considered. As for the 2015 study, all the data came from self-reported measures. Thus, it is possible that participants overestimated their adoption of certain behaviors and that their memory was biased when it came to questions about the actions they undertook during a past flood. In addition, measurement invariance of the indices was validated only for the specific context of the province of Quebec, Canada. The indices would need to be validated again in other countries and cultures before being used in other areas, in order to ensure that the measurement properties of each index can be generalized across different contexts.

## Conclusion

Building upon the foundations established by the creation of the five flood adaptation indices in 2015, this new study succeeded in demonstrating the measurement invariance of the five indices across two different samples of people over time. This substantive methodological work is important for ensuring the improvement of climate change adaptation indices and for providing better measurement of the behavior adoption rates that will be required to adapt to the dangers posed by our changing climate. This assessment of Quebeckers’ flood-related preventive behaviors also showed that, in terms of overall adaptation, little has changed since 2015, despite the occurrence of widely publicized floods between the two measurement periods. This finding highlights a growing need for improved awareness work by health and government agencies.

## Supplementary Information

**Additional file 1.** Standardized factor loadings and uniquenesses of each index. Detailed results for each index regarding the standardized loadings and uniquenesses are available in the Supplemental Materials.

**Additional file 2.** Questionnaire. English version of the questionnaire used in the study.

## Data Availability

The datasets used and/or analyzed during the current study are available from the corresponding author on reasonable request.

## Abbreviations

PTSD: Post-traumatic stress disorder
CFI: Comparative fit index
TLI: Tucker-Lewis index
RMSEA: Root mean square error of approximation

## References

[1] Allard M, Bourque A, Chaumont D, Desjarlais C, Gosselin P, Houle D (2010). Learning to adapt to climate change.

[2] Smith KR, Woodward A, Campbell-Lendrum D, Chadee DD, Honda Y, Liu Q, Field CB, Barros VR, Dokken DJ, Mach KJ, Mastrandrea MD, Bilir TE (2014). Human Health: Impacts, Adaptation, and Co-Benefits. Climate Change 2014: Impacts, adaptation, and vulnerability. Part a:global and Sectoral aspects. Contrubtion of working group II to the fifth assessment report of the intergovernmental panel on climate change.

[3] IPCC (2013). Climate change 2013: the physical science basis. Contribution of working group I to the fifth assessment report of the intergovernmental panel on climate change.

[4] Schar C (2016). Climate extremes: the worst heat waves to come. Nat Clim Chang.

[5] IPCC (2012). Managing the risks of extreme events and disasters to advance climate change adaptation. A special report of working groups I and II of the intergovernmental panel on climate change.

[6] Natural Resources Canada. Climate change in Quebec. 2006. http://ftp.geogratis.gc.ca/pub/nrcan_rncan/publications/ess_sst/212/212816/gscmr_78_f_2001_pr01.pdf. Accessed 12 Oct 2016.

[7] Buttle JM, Allen DM, Caissie D, Davison B, Hayashi M, Peters DL (2016). Flood processes in Canada: regional and special aspects. Can Water Resour J Rev Can Ressour Hydriques.

[8] Normandin P-A. Inondations 2017: Québec attend jusqu’à 245 millions d’Ottawa. La Presse. 2018. https://www.lapresse.ca/actualites/national/201807/12/01-5189360-inondations-2017-quebec-attend-jusqua-245-millions-dottawa.php. Accessed 2 Dec 2019.

[9] Urgence Québec. Bilan provisoire des conséquences - Inondations de 2019. Carrefour de l’information gouvernementale en situation d’urgence. 2019. https://www.urgencequebec.gouv.qc.ca/Fr/CruePrintaniere/Pages/Bilan-provincial-des-cons%C3%A9quences.aspx. Accessed 24 Jun 2019.

[10] Patz JA, Grabow ML, Limaye VS (2014). When it rains, it pours: future climate extremes and health. Ann Glob Health.

[11] Alderman K, Turner LR, Tong SL (2012). Floods and human health: a systematic review. Environ Int.

[12] Confalonieri UEC, Menezes JA, de Souza CM (2015). Climate change and adaptation of the health sector: the case of infectious diseases. Virulence.

[13] Du W, FitzGerald GJ, Clark M, Hou XY (2010). Health impacts of floods. Prehosp Disaster Med.

[14] Lin CJ, Wade TJ, Hilborn ED (2015). Flooding and Clostridium difficile infection: a case-crossover analysis. Int J Environ Res Public Health.

[15] McMichael AJ (2015). Extreme weather events and infectious disease outbreaks. Virulence.

[16] Waite T, Murray V, Baker D. Carbon monoxide poisoning and flooding: changes in risk before, during and after flooding require appropriate public health interventions. PLoS Curr. 2014;6:ecurrents.dis.2b2eb9e15f9b982784938803584487f1. 10.1371/currents.dis.2b2eb9e15f9b982784938803584487f1 PMC4096798 25045587

[17] Chen L, Liu A (2015). The incidence of posttraumatic stress disorder after floods: a meta-analysis. Disaster Med Public Health Prep.

[18] Fernandez A, Black J, Jones M, Wilson L, Salvador-Carulla L, Astell-Burt T (2015). Flooding and mental health: a systematic mapping review. PLoS One.

[19] Lamond JE, Joseph RD, Proverbs DG (2015). An exploration of factors affecting the long term psychological impact and deterioration of mental health in flooded households. Environ Res.

[20] Doocy S, Daniels A, Murray S, Kirsch TD. The human impact of floods: a historical review of events 1980-2009 and systematic literature review. PLoS Curr. 2013;5:ecurrents.dis.f4deb457904936b07c09daa98ee8171a. 10.1371/currents.dis.f4deb457904936b07c09daa98ee8171a PMC3644291 23857425

[21] Acharya MP, Kalischuk RG, Klein KK, Bjornlund H (2007). Health impacts of the 2005 flood events on feedlot farm families in southern Alberta, Canada. Water Resources Management IV.

[22] Hajat S, Ebi KL, Kovats RS, Menne B, Edwards S, Haines A, Kirch W, Bertollini R, Menne B (2005). The human health consequences of flooding in Europe: a review. Extreme weather events and public health responses.

[23] Azuma K, Ikeda K, Kagi N, Yanagi U, Hasegawa K, Osawa H (2014). Effects of water-damaged homes after flooding: health status of the residents and the environmental risk factors. Int J Environ Health Res.

[24] Institut national de santé publique du Québec. Mon climat, ma santé. Inondation: des catastrophes coûteuses. 2010. http://www.monclimatmasante.qc.ca/inondations.aspx.

[25] Gouvernement du Québec. Rapport d’évènement: inondations printanières - Montérégie 2011. 2013. http://www.securitepublique.gouv.qc.ca/fileadmin/Documents/securite_civile/inondations_monteregie_2011/rapport_evenement_inondations_monteregie.pdf.

[26] Statistics Canada. Impact of spring flooding in key areas across Canada. Government of Canada. 2019. https://www150.statcan.gc.ca/n1/en/daily-quotidien/190517/dq190517a-eng.pdf?st=MQDgNCzW. Accessed 30 Mar 2020.

[27] Valois P, Caron M, Gousse-Lessard A-S, Talbot D, Renaud J-S (2019). Development and validation of five behavioral indices of flood adaptation. BMC Public Health.

[28] Cochran WG (1963). Sampling technique.

[29] Millsap RE. Statistical approaches to measurement invariance: Routledge; 2012.

[30] DeVellis RF. Scale Development: Theory and Applications. 4th edition. Los Angeles: SAGE Publications; 2016.

[31] Nunnally J, Bernstein I (1994). Psychometric theory.

[32] Raykov T, Marcoulides GA, Marcoulides GA. Introduction to psychometric theory: Routledge; 2011. 10.4324/9780203841624.

[33] Kent N, Porter J, Dessai S, Miller K, Winne S, Sibille R (2013). PREPARE–the contribution and role of local and household level adaptation in overall UK adaptation. Part of the PREPARE Programme of research on preparedness, adaptation and risk.

[34] Koerth J, Vafeidis A, Carretero S, Sterr H, Hinkel J (2014). A typology of household-level adaptation to coastal flooding and its spatio-temporal patterns. Springerplus.

[35] Kreibich H, Seifert I, Thieken AH, Lindquist E, Wagner K, Merz B (2011). Recent changes in flood preparedness of private households and businesses in Germany. Reg Environ Chang.

[36] Lawrence J, Quade D, Becker J (2014). Integrating the effects of flood experience on risk perception with responses to changing climate risk. Nat Hazards.

[37] Gouvernement du Québec. Inondations - Santé Montérégie. www.santemonteregie.qc.ca. 2008. http://www.santemonteregie.qc.ca/agence/santepublique/directiondesantepublique/inondation.fr.html#.WBnkpy3hBaR. Accessed 5 Sept 2018.

[38] Ville de Saint-Jean-Sur-Richelieu. Les inondations - Que faire avant, pendant et après. www.ville.saint-jean-sur-richelieu.qc.ca. 2018. http://ville.saint-jean-sur-richelieu.qc.ca/securite-civile/Documents/inondations.pdf. Accessed 6 Sept 2018.

[39] Gouvernement du Canada. Inondations. www.preparez-vous.gc.ca. 2015. https://www.preparez-vous.gc.ca/cnt/hzd/flds-fra.aspx. Accessed 5 Sept 2018.

[40] Ministère de la Sécurité publique du Québec. Inondations - Préparons-nous. Sécurité publique Québec. 2018. https://www.securitepublique.gouv.qc.ca/securite-civile/inondation.html. Accessed 5 Sept 2018.

[41] Morin A, Marsh H, Nagengast B. Exploratory structural equation modeling. In: Hancock G, Mueller R, editors. Structural equation modeling: A second course. 2nd ed: IAP; 2013. p. 395–436.

[42] Millsap E (2011). Statistical methods for studying measurement invariance.

[43] Muthén LK, Muthén BO (2015). Mplus User’s Guide.

[44] Byrne BM, Shavelson RJ, Muthén B (1989). Testing for the equivalence of factor covariance and mean structures: the issue of partial measurement invariance. Psychol Bull.

[45] Little TD, Slegers DW, Card NA (2006). A non-arbitrary method of identifying and scaling latent variables in SEM and MACS models. Struct Equ Model.

[46] Litalien D, Lüdtke O, Parker P, Trautwein U (2013). Different pathways, same effects: autonomous goal regulation is associated with subjective well-being during the post-school transition. Motiv Emot.

[47] Ployhart RE, Oswald FL (2004). Applications of mean and covariance structure analysis: integrating correlational and experimental approaches. Organ Res Methods.

[48] Hu L, Bentler PM (1999). Cutoff criteria for fit indexes in covariance structure analysis: conventional criteria versus new alternatives. Struct Equ Model Multidiscip J.

[49] Yu C-Y (2002). Evaluating cutoff criteria of model fit indices for latent variable models with binary and continuous outcomes.

[50] Kline R (2011). Principles and practice of structural equation modeling.

[51] McDonald RP (1999). Test theory : a unified treatment.

[52] Viladrich C, Angulo-Brunet A, Doval E (2017). A journey around alpha and omega to estimate internal consistency reliability. An Psicol Spain.

[53] Urgence Québec. Inondations printanières 2017 - Urgence Québec. 2017. https://www.urgencequebec.gouv.qc.ca/fr/inondation_printanieres_2017/Pages/information-situation.aspx. Accessed 26 Apr 2019.

[54] Radio-Canada.ca IR-CI-. Crue printanière 2019 | Dossier. Radio-Canada.ca. https://ici.radio-canada.ca/dossier/1002429/crue-printaniere-2019-inondations. Accessed 20 May 2020.

[55] Le Devoir. « inondations de 2019» : tous nos articles. https://www.ledevoir.com/motcle/inondations-de-2019. Accessed 20 May 2020.

[56] Henstra D, Minano A, Thistlethwaite J (2019). Communicating disaster risk? An evaluation of the availability and quality of flood maps. Nat Hazards Earth Syst Sci.

[57] IBC (Insurance Bureau of Canada) (2015). The financial management of flood risk: An international review of lessons learned from flood management programs in G8 Countries.

[58] Thistlethwaite J, Henstra D, Brown C, Scott D. Barriers to Insurance as a Flood Risk Management Tool: Evidence from a Survey of Property Owners. Int J Disaster Risk Sci. 2020; 10/gg4bvx.

[59] Ajzen I, Joyce N, Sheikh S, Cote NG (2011). Knowledge and the prediction of behavior: the role of information accuracy in the theory of planned behavior. Basic Appl Soc Psychol.

[60] Alderman K, Turner LR, Tong S (2013). Assessment of the health impacts of the 2011 summer floods in Brisbane. Disaster Med Public Health Prep.

[61] Chen L, Tan H, Cofie R, Hu S, Li Y, Zhou J (2015). Prevalence and determinants of chronic post-traumatic stress disorder after floods. Disaster Med Public Health Prep.

[62] Burton H, Rabito F, Danielson L, Takaro TK (2016). Health effects of flooding in Canada: a 2015 review and description of gaps in research. Can Water Resour J Rev Can Ressour Hydriques.

[63] Waite TD, Chaintarli K, Beck CR, Bone A, Amlôt R, Kovats S (2017). The English national cohort study of flooding and health: cross-sectional analysis of mental health outcomes at year one. BMC Public Health.

[64] Jermacane D, Waite TD, Beck CR, Bone A, Amlôt R, Reacher M (2018). The English National Cohort Study of flooding and health: the change in the prevalence of psychological morbidity at year two. BMC Public Health.

[65] Mulchandani R, Armstrong B, Beck CR, Waite TD, Amlôt R, Kovats S (2020). The English National Cohort Study of Flooding & Health: psychological morbidity at three years of follow up. BMC Public Health.

