# Supplementary material for: Monitoring the evolution of individuals’ flood-related adaptive behaviors over time: two cross-sectional surveys conducted in the Province of Quebec, Canada
Source: BMC Public Health. 2020 Nov 3;20:1643. doi: 10.1186/s12889-020-09763-6 (PMC7607874; doi:10.1186/s12889-020-09763-6)
Supplement: Supplementary file 1 — Additional file 1. Standardized factor loadings and uniquenesses of each index. Detailed results for each index regarding the standardized loadings and uniquenesses are available in the Supplemental Materials. [file 12889_2020_9763_MOESM1_ESM.docx]

**Standardized factor loadings and uniquenesses of each index**

**Standardized factor loadings and uniquenesses of the pre-flood index**

| Behaviors | Factor loadings | Uniquenesses |
| --- | --- | --- |
| Relocate the home elsewhere on the property | 0,376 | 0,859 |
| Make other changes to the building | 0,356 | 0,873 |
| Make other changes to the property to make it more flood-resistant | 0,195 | 0,962 |
| Inquire about the consequences that a flood could have on your physical or mental health | 0,534 | 0,715 |
| Raise the baseboard heaters or electrical outlets on the walls | 0,641 | 0,589 |
| Make a plan for evacuating your neighborhood in case of emergency | 0,414 | 0,829 |
| Replace water-sensitive flooring | 0,636 | 0,595 |
| Inquire about how to better prepare for a flood or to make your home more flood-resistant | 0,635 | 0,597 |
| Waterproof the foundations | 0,656 | 0,570 |
| Make a list of your belongings that could be used for a claim in case of flooding | 0,377 | 0,858 |
| Change the landscape to help water runoff | 0,499 | 0,751 |
| Check to be sure the foundation drain is not blocked | 0,536 | 0,713 |
| Install a backwater valve | 0,586 | 0,657 |
| Own a water pump | 0,473 | 0,776 |
| Know how to cut off the electricity or the water | 0,436 | 0,810 |

**Standardized factor loadings and uniquenesses of the index of adaptation at the time of the alert**

| Behaviors | Factor loadings | Uniquenesses |
| --- | --- | --- |
| Implement other measures to prevent the water from entering (e.g., board up the windows, prepare the water pump, etc.) | 0,312 | 0,903 |
| Block the outside air inlets like the one for the clothes dryer, the range hood, the air exchanger, etc. | 0,78 | 0,391 |
| Waterproof the doors and windows with plastic tape | 0,711 | 0,495 |
| Block the basement drain | 0,461 | 0,788 |
| Cut off the electricity if requested by the authorities | 0,419 | 0,825 |
| Put sandbags on the property | 0,458 | 0,791 |
| Store items or furniture higher or on a higher floor | 0,618 | 0,618 |
| Move your lawn or patio furniture or your vehicle to higher ground | 0,623 | 0,612 |
| Check regularly if the risk of flooding has increased or decreased | 0,221 | 0,951 |

**Standardized factor loadings and uniquenesses of the index during a flood not requiring an evacuation**

| Behaviors | Factor loadings | Uniquenesses |
| --- | --- | --- |
| Wear rubber gloves to handle items in contact with the flood water | 0,770 | 0,407 |
| Boil the water or use bottled water | 0,439 | 0,808 |
| Install a pump to drain the water from the home | 0,499 | 0,751 |
| Wear rubber boots to walk in the flood water | 0,727 | 0,471 |

**Standardized factor loadings and uniquenesses of the index during a flood not requiring an evacuation**

| Behaviors | Factor loadings | Uniquenesses |
| --- | --- | --- |
| Bring your emergency kit, including your medication | 0,269 | 1 |
| Use the route indicated by the authorities to evacuate the neighborhood | 1,973 | 1 |
| Wait for the authorities’ permission before returning home | 0,684 | 1 |
| Tell your loved ones where you can easily be reached | 0,198 | 1 |
| Lock the doors | 0,122 | 1 |

**Standardized factor loadings and uniquenesses of the post-flood index**

| Behaviors | Factor loadings | Uniquenesses |
| --- | --- | --- |
| Check if mold has developed | 0,146 | 0,979 |
| Replace the refrigerator insulation if it is wet or replace the appliance | 0,485 | 0,765 |
| Attend citizens’ meetings concerning the flood | 0,614 | 0,623 |
| Update your emergency kit | 0,196 | 0,962 |
| Sterilize all kitchen items contaminated by the flood water | 0,693 | 0,519 |
| Discard items in contact with the flood water | 0,781 | 0,391 |
| Have the condition of the electrical installation and heating appliances checked | 0,76 | 0,423 |
| Wear rubber gloves to handle items in contact with the flood water | 0,608 | 0,630 |
| Make a list of the damages caused to the home and to your belongings | 0,775 | 0,400 |
| Disinfect the contaminated rooms | 0,776 | 0,398 |
