# Supplementary material for: Monitoring the evolution of individuals’ flood-related adaptive behaviors over time: two cross-sectional surveys conducted in the Province of Quebec, Canada
Source: BMC Public Health. 2020 Nov 3;20:1643. doi: 10.1186/s12889-020-09763-6 (PMC7607874; doi:10.1186/s12889-020-09763-6)
Supplement: Supplementary file 2 — Additional file 2. Questionnaire. English version of the questionnaire used in the study. [file 12889_2020_9763_MOESM2_ESM.docx]

**Questionnaire**

1. **CM1**

Including yourself, how many people live at this address? *Note to the interviewer:* *make sure the respondent counted everyone, including people who usually live at this address but are currently absent, such as students who come back to live with their parents, spouses who work out of town and those temporarily hospitalized.*

WRITE THE NUMBER OF PERSONS: ______

Lives alone 01 => QUA1

DNK 98

Refusal 99

1. **CM2**

Which of the following types of households best describes your current situation?

You live as a couple, but with no children 01 => QUA1

You live as a couple with one or more children (biological or

not) 02

You live alone with one or more children (biological or not) 03

You live with one or more persons who are not related to you

(e.g. co-tenant) 04

You live in another type of household 05

DNK 98

Refusal 99

1. **CM3**

If “CM2 = 02-03-04-05,” among the children who live at this address, how many are…? *Note to the interviewer:* *they could be children who do not belong to the respondent, such as grandchildren living with their grandparents or those of a co-tenant.*

READ OUT THE CATEGORIES AND WRITE THE NUMBER OF CHILDREN PER STRATUM

From 0 to 5 years old: ________

From 6 to 12 years old: ________

From 13 to 17 years old: ________

18 years old and over: ________

No children: 97

DNK 98

Refusal 99

The next questions concern the neighborhood and the home where you live. By “neighborhood,” we mean anything within a 15- to 20-minute walk from your home, for a person walking at a steady, normal pace.

1. **QUA1**

In all, how many years have you lived in this neighborhood?

WRITE THE DURATION IN YEARS HERE: ______ OR “00” IF LESS THAN ONE YEAR

Less than one year 00

DNK 98

Refusal 99

1. **QUA2**

How many more years do you plan on living in this neighborhood?

WRITE THE NUMBER OF YEARS HERE: ___________ OR “OO” IF LESS THAN 1 YEAR

Less than 1 year 00

DNK 98

Refusal 99

1. **QUA3**

Not taking into account the renovations you want to do to it, would you say that your home:

READ OUT THE CHOICES

Satisfies your needs 01

Mostly satisfies your needs 02

Doesn’t really satisfy your needs 04

Doesn’t satisfy your needs at all 05

DNK 98

Refusal 99

1. **QUA4**

How would you describe your sense of belonging to your local community? Would you say that it is:

READ OUT THE CHOICES

Very strong 01

Rather strong 02

Rather weak 03

Very weak 04

DNK 98

Refusal 99

1. **QUA5**

To your knowledge, do you live in a flood-risk zone?

SPONTANEOUS ANSWER

*Note to the interviewer: if the person seems disturbed by the question, you can reassure her by telling her that: this is not a political survey, its only goal is to better inform us on the adaptive behaviors that can be adopted by the population. You can go on the website* [*www.oqacc.com*](http://WWW.OQACC.COM) *to see a list of the Observatoire’s current research projects and a list of health resource if you need support.*

Yes 01 => QUA6

No 02 => LOG1

DNK 98 => LOG1

Refusal 99 => LOG1

1. **QUA6**

If “QUA3 = 01,” do you know the flood elevation of the zone:

READ OUT THE CHOICES

*Note to the interviewer: if the person asks you to explain the meaning of flood elevation, you can say that:*
*0 to 20 years means that each year, there is more than a 5% chance that the territory will be flooded*
*20 to 100 years means that each year, there is between a 1% and a 5% chance that the territory will be flooded*
*100 years or more means that each year, there is less than 1% chance that the territory will be flooded*

0 to 20-year flood elevation 01 => QUA7

20 to 100- year flood elevation 02 => QUA7

100-year flood elevation or more 03 => QUA7

DNK 98 => LOG1

Refusal 99 => LOG1

1. **QUA7**

If “QUA4 = 01, 02 or 03,” which source(s) did you consult to obtain this information?

READ OUT THE CHOICES

Municipality / Map of flood-risk zones 01

Your neighbors 02

Your family or friends 03

Your own judgment 04

Other. Please specify 05

DNK 98

Refusal 99

1. **LOG1**

How long have you lived in this home? *Note to the interviewer: by “home,” we mean any type of residence where a household lives.*

WRITE THE NUMBER OF YEARS: ______ OR “00” IF LESS THAN A YEAR

Less than a year 00

DNK 98

Refusal 99

1. **LOG2**

Do you or a member of your household own this home?

Yes (owner) 01

No (tenant) 02

DNK 98

Refusal 99

1. **LOG3**

In which type of home do you live? Is it:

READ OUT THE CHOICES

A house (including: townhouse, semi-detached, single-family) 01 => LOG5

A mobile home 02 => LOG5

An apartment or a condo building of four stories or less 03 => LOG4A

An apartment or a condo building of five stories or more 04 => LOG4A

Other (e.g. hotel, rooming house, boarding house) 05 => LOG5

DNK 98 => LOG5

Refusal 99 => LOG5

1. **LOG4A**

If “LOG3 = 03 or 04,” on which floor is your home located?

*Note to the interviewer: for respondents who say “1st floor,” make sure that they mean the ground floor*

WRITE THE FLOOR HERE: _______ => LOG5

DNK 98 => LOG4B

Refusal 99 => LOG4B

1. **LOG4B**

If “LOG4A = DNK or Refusal,” is your home located___?

READ OUT THE CHOICES

On a top floor 01

On a middle floor 02

On a lower floor 03

DNK 98

Refusal 99

1. **LOG5**

Do you have access to a basement in your home? Note that by “basement,” we mean any room or floor that is entirely or partly below ground level. A crawl space is not considered a basement.

Yes 01

No 02 => LOG7

DNK 98 => LOG7

Refusal 99 => LOG7

1. **LOG6**

If “LOG5 = 01,” what is the level of finishing of the basement to which you have access? Would you say that it is:

READ OUT THE CHOICES

Fully finished (i.e. the walls and ceilings are closed, the floor is

covered) and the rooms are furnished 01

Partly finished (i.e. part of the walls and ceilings are closed and the

floor is partly covered) and part of the rooms are furnished 02

Not finished (e.g. an apartment locker) 03

DNK 98

Refusal 99

1. **LOG7**

Disregarding the renovations or additions that you would like, would you say that your home needs:

Only regular maintenance, such as painting and cleaning the

heating system 01

Minor repairs, such as lifting or missing floor tiles, torn-off shingles,

handrails or defective exterior finishing 02

Major repairs, such as plumbing or defective electrical installation,

or repairs to the wall, floor or ceiling structures 03

DNK 98

Refusal 99

1. **LOG8**

Does your water come from a municipal or private water system or from an individual well?

Individual well 01

Water system (municipal or private) 02

DNK 98

Refusal 99

1. **PERC00**

I will now ask you a few questions about floods. But first, it is important to specify that we are concerned only with floods coming from a watercourse, such as a river overflowing due to rapid melting of the snow or heavy rains. For our study, sewers backing up, broken pipes or aqueducts WITHOUT flooding from a watercourse are not considered to be floods.

1. **RISQ1**

In your opinion, what is the risk of your current home being flooded in the next five years? Would you say that it is:

Very high 01

High 02

Moderate 03

Low 04

Very low 05

Nil 06

DNK 98

Refusal 99

1. **SEV1**

Some people who are flooded say they feel negative impacts on their physical or mental health. If you have been a victim of flooding, would you say that the negative consequences for your physical health would be:

READ OUT THE CHOICES

Very severe 01

Quite severe 02

Not very severe 03

No negative consequence 04

DNK 98

Refusal 99

1. **SEV2**

And would you say that the negative consequences for your mental health would be:

READ OUT THE CHOICES

Very severe 01

Quite severe 02

Not very severe 03

No negative consequence 04

DNK 98

Refusal 99

1. **SEV3**

If you have been a victim of flooding, would you say that the damage caused to your home and your personal effects would be:

READ OUT THE CHOICES

Very severe 01

Quite severe 02

Not very severe 03

No damages 04

DNK 98

Refusal 99

1. **EXP1**

Since you’ve lived in your current home, has your municipality ever issued a flood alert that concerned you? Note that by “flood alert,” we mean that the municipality informs its citizens that there is a risk of flooding in the days following the message.

Yes 01

No 02 => EXP3

DNK 98 => EXP3

Refusal 99 => EXP3

1. **EXP2**

When was the last flood alert that you experienced in your current home? *Note to the interviewer: ask the respondent to specify the year and the month. If he/she no longer remembers the month, ask him/her minimally during which season the last flood alert that he/she experienced occurred and if he/she no longer remembers the year, ask him/her to answer according to the intervals proposed.*

WRITE THE YEAR HERE: _______

DNK 98

Refusal 99

If HE/SHE DOESN’T KNOW THE YEAR OR REFUSES, ASK FOR AN INTERVAL:

Less than 2 years 01

From 2 to 4 years 02

From 5 to 10 years 03

More than 10 years 04

DNK 98

Refusal 99

WRITE THE MONTH HERE: _______

DNK 98

Refusal 99

If HE/SHE DOESN’T KNOW THE MONTH OR REFUSES, ASK FOR THE SEASON:

Summer (June 21 to September 20) 01

Fall (September 21 to December 20) 02

Winter (December 21 to March 20) 03

Spring (March 21 to June 20) 04

DNK 98

Refusal 99

1. **EXP3**

How many times have you been flooded in your current home?

Never 00 => EXP5

1 time 01

2 times 02

3 times 03

4 times 04

5 times or more 05

DNK 98 => EXP5

Refusal 99 => EXP5

1. **EXP4**

When was the last flood that you experienced in your current home? *Note to the interviewer: ask the respondent to specify the year and the month. If he/she no longer remembers the month, ask him/her minimally during which season the last flood that he/she experienced occurred and if he/she no longer remembers the year, ask him/her to answer according to the intervals proposed.*

WRITE THE YEAR HERE: _______

DNK 98

Refusal 99

If HE/SHE DOESN’T KNOW THE YEAR OR REFUSES, ASK FOR AN INTERVAL:

Less than 2 years 01

From 2 to 4 years 02

From 5 to 10 years 03

More than 10 years 04

DNK 98

Refusal 99

WRITE THE MONTH HERE: _______

DNK 98

Refusal 99

If HE/SHE DOESN’T KNOW THE MONTH OR REFUSES, ASK FOR THE SEASON:

Summer (June 21 to September 20) 01

Fall (September 21 to December 20) 02

Winter (December 21 to March 20) 03

Spring (March 21 to June 20) 04

DNK 98

Refusal 99

1. **EXP5**

How many times have you experienced a flood in a home other than the one where you currently live?

Never 00 => EXP7

1 time 01

2 times 02

3 times 03

4 times 04

5 times or more 05

DNK 98 => EXP7

Refusal 99 => EXP7

1. **EXP6**

When was the last flood you experienced in a home other than the one where you currently live?

READ OUT THE CATEGORIES

Less than 2 years 01

Between 2 and 4 years 02

Between 5 and 10 years 03

More than 10 years 04

DNK 98

Refusal 99

1. **EXP7**

If “EXP3 = 01, 02, 03, 04, 05,” thinking about the flood that you experienced in (SAY THE DATE GIVEN IN “EXP4”), did the water:

READ OUT THE CHOICES

Enter your home 01

Remain on the ground around your home 02 => EXP11

Remain in the street without reaching your property 03 => EXP11

DNK 98 => EXP11

Refusal 99 => EXP11

1. **EXP8A**

If “EXP7 = 01,” and what was the approximate water level inside your home? *Note to the interviewer: if the respondent is unable to estimate the water level in the proposed units, use a body reference, such as ankle, knee, waist or shoulder deep, or above the head.*

WRITE THE UNIT USED BY THE RESPONDENT HERE

Inches 01

Feet 02

Centimeters 03

Meters 04

DNK 98

Refusal 99

1. **EXP8B**

And what was the approximate water level inside your home?

WRITE THE QUANTITY OR BODY REFERENCE HERE: _________

DNK 98

Refusal 99

1. **EXP9**

If “EXP7 = 01,” still thinking about the flood that you experienced in (SAY THE DATE GIVEN IN “EXP4”), approximately how many DAYS did the water remain in your home? *Note to the interviewer: if the respondent gives the answer in weeks or months, convert it to a number of days.*

WRITE THE NUMBER OF DAYS: ______ OR “01” IF ONE DAY OR LESS.

One day or less 01

DNK 98

Refusal 99

1. **EXP10**

If “EXP3 = 01, 02, 03, 04, 05,” still thinking about the flood that you experienced in (SAY THE DATE GIVEN IN “EXP4”), did the authorities ask you to evacuate your home?

Yes 01

No 02 => PRE1

DNK 98 => PRE1

Refusal 99 => PRE1

1. **EXP11**

If “EXP10 = 01,” and did you evacuate your home further to the authorities’ request?

Yes 01

No 02 => PRE1

DNK 98 => PRE1

Refusal 99 => PRE1

1. **EXP12**

If “EXP11 = 01,” after this evacuation, approximately how many DAYS did you have to live away from home? *Note to the interviewer: if the respondent gives the answer in weeks or months, convert it to a number of days.*

WRITE THE NUMBER OF DAYS HERE: ________

DNK 98

Refusal 99

1. **PRE1**

The next questions concern preventive measures that some people take to better prepare for a flood or to make their home more water-resistant. Do you have a list of telephone numbers that could be useful in case of an emergency?

Yes 01

No 02 => PRE3

DNK 98 => PRE3

Refusal 99 => PRE3

1. **PRE2**

Does your list include the following numbers?

READ OUT THE CHOICES AND WRITE YES\NO\DNK\REFUSAL FOR EACH ONE

Treating physician: ________

Pharmacist: ________

Hydro-Québec: _______

Québec’s Poison Control Centre: _________

Municipality: ________

A family member or friend who lives in your neighborhood or a neighbor: ________

Other? Please specify: ________

1. **PRE3**

Do you have an emergency kit to fill your essential needs for 72 hours in case of a flood? This kit should contain a battery-run radio, replacement batteries, a flashlight, candles, a lighter or matches, a first-aid kit, two liters of water per person per day, canned foods, a manual can opener, warm blankets and your prescription mediation.

Yes, it has most of these items 01

Yes, but it has only some of these items 02

No, I don’t have a kit 03

DNK 98

Refusal 99

1. **PRE4**

Have you made a list of your belongings that could be used for a claim in case of flooding?

Yes 01

No 02 => PRE6

DNK 98 => PRE6

Refusal 99 => PRE6

1. **PRE5**

If “PRE4 = 01,” and have you documented the list of your belongings using photos or videos?

Yes 01

No 02

DNK 98

Refusal 99

1. **PRE6**

Have you made a plan for evacuating your home in case of emergency, notably by identifying the emergency exits and designating an outdoor meeting place for your family members?

Yes 01

No 02

DNK 98

Refusal 99

1. **PRE7**

Have you made a plan for evacuating your neighborhood in case of emergency, notably by planning an alternative evacuation route from your neighborhood in case the usual route is impassable?

Yes 01

No 02

DNK 98

Refusal 99

1. **PRE8A**

In your home, do you have access to the main water valve?

Yes 01 => PRE8B

No 02 => PRE9A

DNK 98 => PRE9A

Refusal 99 => PRE9A

1. **PRE8B**

If “PRE8A = 01,” in case of flooding, would you know how to shut off the water?

Yes 01

No 02

DNK 98

Refusal 99

1. **PRE9A**

In your home, do you have access to the electrical panel?

Yes 01 => PRE9B

No 02 => PRE10A

DNK 98 => PRE10A

Refusal 99 => PRE10A

1. **PRE9B**

If “PRE9A = 01,” in case of flooding, would you know how to cut off the electricity?

Yes 01

No 02

DNK 98

Refusal 99

1. **PRE10A**

In your home, do you have access to the natural gas shutoff valve?

Yes 01 => PRE10B

No 02 => PRE11

N/A 03 => PRE11

DNK 98 => PRE11

Refusal 99 => PRE11

1. **PRE10B**

If “PRE10A = 01,” in case of flooding, would you know how to shut off the natural gas?

Yes 01

No 02

DNK 98

Refusal 99

1. **PRE11**

Have you ever inquired about how to better prepare for a flood or to make your home more flood-resistant?

Yes 01 => PRE12

No 02 => PRE13

DNK 98 => PRE13

Refusal 99 => PRE13

1. **PRE12**

If “PRE11 = 01,” which information source(s) did you consult?

READ OUT THE CHOICES (MORE THAN ONE POSSIBLE ANSWER).

Your municipality 01

The Québec government’s website 02

The Canadian government’s website 03

Other Internet sites 04

Your neighbors 05

Your friends or family 06

Other. Please specify 07

DNK 98

Refusal 99

1. **PRE13**

Have you ever inquired about the consequences that a flood could have on your physical or mental health?

Yes 01 => PRE14

No 02 => PRE15

DNK 98 => PRE15

Refusal 99 => PRE15

1. **PRE14**

If “PRE13 = 01,” which information source(s) did you consult?

READ OUT THE CHOICES (MORE THAN ONE POSSIBLE ANSWER).

The newspapers 01

The radio 02

The television 03

The Québec government’s website 04

The Canadian government’s website 05

Your neighbors 06

Your friends or family 07

Other. Please specify 08

DNK 98

Refusal 99

1. **PRE15**

If “LOG2 = 01,” since you’ve owned this home, to make your home more flood-resistant, have you ever done or had someone do…? If “LOG2 = 02,” since you’ve lived in this home, has the owner of the building ever done or had someone do…:

READ OUT THE CHOICES AND WRITE YES/NO/DNK/REFUSAL FOR EACH ONE

Waterproof the foundations: _________

Raise the door sills: _________

Raise the foundations (including the installation of pilings): _________

Raise the baseboard heaters and electrical outlets on the walls: __________

Replace water-sensitive flooring (e.g. carpeting) with a waterproof finish (e.g. ceramic): __________

Install a backwater valve: __________

Other? Please specify: __________

1. **PRE16**

If “LOG2 = 01,” since you’ve owned this home, to make your property more flood-resistant, have you ever done or had someone do…? If “LOG2 = 02,” since you’ve lived in this home, has the owner of the building done or had someone do…:

READ OUT THE CHOICES AND WRITE YES/NO/DNK/REFUSAL FOR EACH ONE

Reduce the amount of surfaces that are not waterproof (e.g. replace asphalt with stones or another finish that lets the water through: _________

Change the landscape to help water runoff: _________

Drainage work around the home: _________

Check to be sure the foundation drain is not blocked: __________

Other? Please specify: __________

1. **PRE17**

If “LOG2 = 01,” do you own a water pump that you could use to remove the water from your home in case of a flood?

Yes 01

No 02 => PRE18

DNK 98 => PRE18

Refusal 99 => PRE18

1. **PRE17b**

Is your water pump readily accessible?

Yes 01

No 02

DNK 98

Refusal 99

1. **PRE18**

If “LOG5 = 01,” in the basement, do you store valuable items that could be severely damage in a flood? *Note to the interviewer: specify that by “valuable items,” we mean items that have monetary value (e.g. television) and non-monetary (e.g. souvenirs, photos, important documents) value.*

Yes 01

No 02

DNK 98

Refusal 99

1. **PRE19**

Have you done anything else that we did not mention to better prepare for a flood or to make your home more flood-resistant?

SPONTANEOUS ANSWER FROM THE RESPONDENT – MAXIMUM OF 3 THINGS, IN ORDER OF IMPORTANCE (THE FIRST MENTIONED BEING THE MOST IMPORTANT).

Yes. Please specify: __________________________________

No 02

DNK 98

Refusal 99

1. **PRE20**

If “EXP3 = 01, 02, 03, 04, 05,” when you think about the various things that you’ve adopted to protect your home and make it more flood-resistant (e.g. get a water pump, etc.), would you say that you had completed them before the flood that you experienced in (SAY THE DATE GIVEN IN “EXP4”)?

READ OUT THE CHOICES OF ANSWERS

Yes, I had completed the majority of them before the

last flood 01

Yes, but I had adopted only a few of them before the last flood 02

No, I did them after the last flood to be better prepared in the

future 03

DNK 98

Refusal 99

1. **PRE21**

If “EXP3 = 01, 02, 03, 04, 05,” when you think about the various things you adopted to protect your safety and your health (e.g. get an emergency kit, etc.), would you say that you had already completed them before the flood that you experienced in (SAY THE DATE GIVEN IN “EXP4”)?

READ OUT THE CHOICES OF ANSWERS

Yes, I had already completed most of them
before the last flood 01

No, I did them during the last flood 02

No, I did them after the last flood to be better prepared in the

future 03

DNK 98

Refusal 99

1. **ALT00**

If “EXP1 = 01,” the next questions concern the period of alert that precedes a flood, that is, when the municipality issues a flood alert to its citizens. To answer the questions, you must think about the flood alert that you experienced in (SAY THE DATE GIVEN IN “EXP2”).

1. **ALT1**

Thinking about the flood alert experienced in (SAY THE DATE GIVEN IN “EXP2”), did you move your lawn or patio furniture and your car to higher ground?

Yes 01

No 02

N/A 03

DNK 98

Refusal 99

1. **ALT2**

Thinking about the flood alert experienced in (SAY THE DATE GIVEN IN “EXP2”), did you place as many items as possible, such as furniture, souvenirs or important documents, higher up or on the upper floor?

Yes 01

No 02

DNK 98

Refusal 99

1. **ALT3**

If “LOG2 = 01” **AND** if “LOG5 = 01,” did you block your basement drain during the flood alert experienced in (SAY THE DATE GIVEN IN “EXP2”)?

Yes 01

No 02

I have a backwater valve 03

DNK 98

Refusal 99

1. **ALT4**

During the flood alert and when requested by the authorities, did you shut off:

READ OUT THE CHOICES AND WRITE YES/NO / NOT APPLICABLE / DNK FOR EACH ONE

The gas: ___________

The electricity: _________

1. **ALT5**

Still thinking about the flood alert that you experienced in (SAY THE DATE GIVEN IN “EXP2”), which of the following measures that apply to your situation did you take to prevent the water from coming into your home:

READ OUT THE CHOICES AND WRITE YES/NO/DNK/REFUSAL FOR EACH ONE

Seal the doors and windows with plasticized adhesive tape: __________

Block the outside air inlets, such as those for the dryer, the range hood, the air exchanger, etc.: __________

Lay sandbags on the ground: ___________

Other. Please specify: ___________

1. **ALT6**

Still during the alert experienced in (SAY THE DATE GIVEN IN “EXP2”), did you check regularly to find out if the risk of flooding had increased or decreased?

Yes 01 =>ALT7

No 02 =>ALT8

DNK 98 =>ALT8

Refusal 99 =>ALT8

1. **ALT7**

If “ALT6 = 01,” which information source(s) did you consult to find out about the flood risk?

READ OUT THE CHOICES (MORE THAN ONE POSSIBLE ANSWER).

The radio 01

The television 02

The Internet 03

Printed newspapers 04

A text alert system 05

Neighbors, friends or families 06

Your own judgment 07

Other. Please specify 07

DNK 98

Refusal 99

1. **ALT8**

Did you or another person with whom you live help your neighbors take protective measures, such as laying out sandbags?

Yes 01

No 02

No nearby neighbors 03

DNK 98

Refusal 99

1. **ALT9**

Still thinking about the flood alert that you experienced in (SAY THE DATE GIVEN IN “EXP2”), are there any other things you did to prepare for the flood?

SPONTANEOUS ANSWER FROM THE RESPONDENT – MAXIMUM OF 3 THINGS, IN ORDER OF IMPORTANCE (THE FIRST MENTIONED BEING THE MOST IMPORTANT).

Yes. Please specify: __________________

No 02

DNK 98

Refusal 99

1. **EVAC00**

If “EXP11 = 01,” the next questions concern the flood that you experienced in (SAY THE DATE GIVEN IN “EXP4”). They pertain more specifically to your behavior when you evacuated your home.

1. **EVAC1**

If “PRE3 = 01,” when you were evacuating, did you bring your emergency kit, including your medication if you take any?

Yes 01

No 02

DNK 98

Refusal 99

1. **EVAC2**

Did you lock your doors when you left your home?

Yes 01

No 02

DNK 98

Refusal 99

1. **EVAC3**

Was a temporary shelter made available to you?

Yes 01

No 02

DNK 98

Refusal 99

1. **EVAC4**

If “EVAC3 = 01,” and did you register at this temporary shelter?

Yes 01

No 02

DNK 98

Refusal 99

1. **EVAC5**

Did you use the route indicated by the authorities to evacuate your neighborhood?

Yes 01

No 02

I was not aware of this information 03

DNK 98

Refusal 99

1. **EVAC6**

Did you tell a family member or a friend where you could be easily reached after the evacuation?

Yes 01

No 02

DNK 98

Refusal 99

1. **EVAC7**

If you could not bring your pets with you, did you put them in a secure location before evacuating your home?

Yes, all of them 01

Yes, some of them 02

No 03

I have no pets 04

DNK 98

Refusal 99

1. **EVAC8**

Still thinking about the flood that you experienced in (SAY THE DATE GIVEN IN “EXP4”) and more specifically about the point where the authorities asked you to evacuate your home, did you do anything else to ensure your safety or to protect your home before evacuating?

SPONTANEOUS ANSWER FROM THE RESPONDENT – MAXIMUM OF 3 THINGS, IN ORDER OF IMPORTANCE (THE FIRST MENTIONED BEING THE MOST IMPORTANT).

Yes. Please specify: _________________

No 02

DNK 98

Refusal 99

1. **INON00**

If “EXP10 = 02” **OR** “EXP11 = 02,” the next questions still concern the flood you experienced in (SAY THE DATE GIVEN IN “EXP4”). They pertain more specifically to your behavior during the flood.

1. **INON1**

If “LOG8 = 02,” did you boil the tap water or use bottled water until the municipality or the water system operator confirmed that the water was safe to drink **OR** if “LOG8 = 01,” did you boil your water or use bottled water until an analysis of the water quality confirmed that the water was safe to drink?

Yes 01

No 02

DNK 98

Refusal 99

1. **INON2**

During the flood of (SAY THE DATE GIVEN IN “EXP4”), how often did you wear rubber gloves to handle objects that were in contact with flood water?

Always 01

Most of the time 02

Sometimes 03

Never 04

DNK 98

Refusal 99

1. **INON3**

During the flood of (SAY THE DATE GIVEN IN “EXP4”), how often did you wear rubber boots to walk in the flood water?

Always 01

Most of the time 02

Sometimes 03

Never 04

DNK 98

Refusal 99

1. **INON4**

Still thinking about the flood of (SAY THE DATE GIVEN IN “EXP4”), did you do anything else to ensure your safety or to protect your home and your belongings?

SPONTANEOUS ANSWER FROM THE RESPONDENT – MAXIMUM OF 3 THINGS, IN ORDER OF IMPORTANCE (THE FIRST MENTIONED BEING THE MOST IMPORTANT).

Yes. Please specify 01

No 02

DNK 98

Refusal 99

1. **POST00A**

If “EXP11 = 01,” the last questions in this section still concern the last flood you experienced, in (SAY THE DATE GIVEN IN “EXP4”), and more specifically the moment when you could return to your home and start the clean-up.

1. **POST00B**

If “EXP10 = 02” or “EXP11 = 02,” the last questions in this section still concern the last flood that you experienced, in (SAY THE DATE GIVEN IN “EXP4”), and pertain more specifically to the period after the flood, that is, when the water receded and you could start the clean-up.

1. **POST1**

If “EXP11 = 01,” during the flood of (SAY THE DATE GIVEN IN “EXP4”), did you wait for the authorities’ permission before returning home?

Yes 01

No 02

DNK 98

Refusal 99

1. **POST2**

If “EXP7 = 01,” after the flood, did you have a specialist check the condition of the electrical installation and heating appliances?

Yes 01 => **POST3**

No 02 => **POST2b**

DNK 98 => **POST3**

Refusal 99 => **POST3**

1. **POST2b**

What is the main reason why you did not get a specialist to check the condition of the wiring and heating appliances?

WRITE THE ANSWER: ___________________________________

DNK 98

Refusal 99

1. **POST3**

If “EXP7 = 01,” if the inside of your hot water tank, fridge or freezer got wet, did you have their insulation replaced or did you replace the appliance if it was too badly damaged?

Yes 01

No 02

DNK 98

Refusal 99

1. **POST4**

If “EXP11 = 01” **AND** “LOG8 = 02,” did you confirm with your municipality or your water system operator that the water was safe to drink before consuming it **OR** if “EXP11 = 01” **AND** “LOG11 = 01,” did you have your well water analyzed before drinking it?

Yes 01

No 02

DNK 98

Refusal 99

1. **POST5**

If “EXP7 = 01,” during the clean-up phase, did you make sure to disinfect or have someone disinfect the rooms in your home that were contaminated by the flood water?

Yes 01 => **POST6**

No 02 => **POST5b**

DNK 98 => **POST6**

Refusal 99 => **POST6**

1. **POST5b**

What is the main reason why you did not disinfect the rooms of your home that were contaminated by the flood water or have them disinfected?

WRITE THE ANSWER: ___________________________________

DNK 98

Refusal 99

1. **POST6**

If “EXP7 = 01,” during the clean-up phase, did you make sure to sterilize, according to the instructions received, all kitchen items that were contaminated by flood water? *Note to the interviewer: the items must really have been sterilized with boiling water or another sterile solution. If the respondent says he/she only washed them with dish soap, write “No.”*

Yes 01

No 02

DNK 98

Refusal 99

1. **POST7**

If “EXP7 = 01,” several items that were in direct contact with flood water must be discarded because they cannot be properly cleaned. This is the case, for example, of perishable and non-perishable foods, medication, toiletries (such as make-up, razor, tooth brush, etc.), mattresses and box springs, pillows and upholstered furniture, carpets, wool insulation, wall paper, etc. After the last flood, would you say that you discarded:

READ OUT THE CHOICES

All of these items 01

Most of these items 02

A few of these items 03

None of these items 04

DNK 98

Refusal 99

1. **POST8**

If “EXP7 = 01, 02,” during the clean-up phase, how often did you wear rubber gloves to handle items that had been in contact with flood water?

READ OUT THE CHOICES

Always 01

Most of the time 02

Sometimes 03

Never 04

DNK 98

Refusal 99

1. **POST9**

If “EXP7 = 01,” in the weeks and months after the water had receded, did you notice any mold or mildew developing inside your home? Note that mold and mildew show up as colored or blackish stains on the surface of materials and smell musty, earthy or of alcohol.

Yes 01

No 02

DNK 98

Refusal 99

1. **POST10**

If “EXP7 = 01, 02,” did you make a list of the damages caused to your home and material property by the flood?

Yes 01

No 02 =>POST12

DNK 98 =>POST12

Refusal 99 =>POST12

1. **POST11**

If “POST10 = 01,” and did you take photos or videos to document the damage?

Yes 01

No 02

DNK 98

Refusal 99

1. **POST12**

If “PRE3 = 01,” did you update your emergency kit after the flood?

Yes 01

No 02

No, I didn’t use it 03

DNK 98

Refusal 99

1. **POST13**

In the year following the flood that you experienced in (SAY THE DATE GIVEN IN “EXP4”), approximately how many times did you participate in citizen meetings relating to the flood? For example, meetings organized by your municipality, public security or a citizen group.

WRITE THE NUMBER OF TIMES HERE: __________

DNK 98

Refusal 99

1. **POST14**

Still thinking about the period after the flood of (SAY THE DATE GIVEN IN “EXP4”), did you do anything else to ensure your safety or to reduce the risks for your physical or mental health?

SPONTANEOUS ANSWER FROM THE RESPONDENT – MAXIMUM OF 3 THINGS, IN ORDER OF IMPORTANCE (THE FIRST MENTIONED BEING THE MOST IMPORTANT).

Yes. Please specify: _______________________

No 02

DNK 98

Refusal 99

1. **IMP00**

If “EXP3 = 01, 02, 03, 04, 05,” the next questions concern the impacts that the flood of (SAY THE DATE GIVEN IN “EXP4”) had on your health and your home. Please remember that all your answers will remain strictly confidential.

1. **IMP1**

Some people say that floods affect their physical health, mental health or both. Was your physical health negatively affected by the flood of (SAY THE DATE GIVEN IN “EXP4”)? Would you say:

READ OUT THE CHOICES

Very much 01 => IMP2

Moderately 02 => IMP2

Slightly 03 => IMP4

Not at all 04 => IMP4

DNK 98 => IMP4

Refusal 99 => IMP4

1. **IMP2**

If “IMP1 = 01 or 02,” what were these physical health problems?

Write down the problem(s). Maximum of 3, in order of importance (the first mentioned being the most important).

1:__________________

2:__________________

3:__________________

DNK 98

Refusal 99

1. **IMP3**

If “IMP1 = 01, 02,” did you consult a health professional, such as a doctor, physiotherapist or chiropractor, because of one of these physical health problems?

Yes 01

No 02

DNK 98

Refusal 99

1. **IMP4**

And was your mental health negatively affected by the flood of (SAY THE DATE GIVEN IN “EXP4”)? Would you say:

READ OUT THE CHOICES

Very much 01 => IMP5

Moderately 02 => IMP5

Slightly 03 => IMP7

Not at all 04 => IMP7

DNK 98 => IMP7

Refusal 99 => IMP7

1. **IMP5**

If “IMP4 = 01 or 02,” what were these mental health problems?

Write down the problem(s). Maximum of 3, in order of importance (the first mentioned being the most important).

1:__________________

2:__________________

3:__________________

DNK 98

Refusal 99

1. **IMP6**

If “IMP4 = 01 or 02,” did you consult a health professional, such as a doctor, a psychologist, a psychotherapist, a nurse or a social worker because of the consequences of the flood for your mental well-being?

Yes 01

No 02

DNK 98

Refusal 99

1. **IMP7**

**If “EXP7 = 01-02,”** still thinking about the flood of (SAY THE DATE GIVEN IN “EXP4”), at how much do you estimate in total the material damages caused? Note that by “material damages,” we mean damages both to your home if you are the owner and to your material belongings, such as your furniture and personal effects.

No damage 01 => IMP8

Less than $5,000 02

Between $5,001 and $10,000 03

Between $10,001 and $15,000 04

Between $15,001 and $20,000 05

Between $20,001 and $25,000 06

Between $25,001 and $30,000 07

Between $30,001 and $35,000 08

Between $35,001 and $40,000 09

Between $40,001 and $45,000 10

Between $45,001 and $50,000 11

Between $50,001 and $55,000 12

Between $55,001 and $60,000 13

Between $60,001 and $65,000 14

Between $65,001 and $70,000 15

Between $70,001 and $75,000 16

Between $75,001 and $100,000 17

More than $100,000 18

DNK 98 => IMP8

Refusal 99 => IMP8


**IMP7B**

**If “EXP7 = 01-02,”** Have you received compensation for these material damages from the government?

Yes 01 => IMP7C

No 02 => IMP7D

DNK 98 => IMP7D

Refusal 99 => IMP7D

1. **IMP7C**

**If “EXP7 = 01-02,”** If you do not mind, could you tell us approximately how much was this compensation in dollars?

Less than $5,000 02

Between $5,001 and $10,000 03

Between $10,001 and $15,000 04

Between $15,001 and $20,000 05

Between $20,001 and $25,000 06

Between $25,001 and $30,000 07

Between $30,001 and $35,000 08

Between $35,001 and $40,000 09

Between $40,001 and $45,000 10

Between $45,001 and $50,000 11

Between $50,001 and $55,000 12

Between $55,001 and $60,000 13

Between $60,001 and $65,000 14

Between $65,001 and $70,000 15

Between $70,001 and $75,000 16

Between $75,001 and $100,000 17

More than $100,000 18

DNK 98

Refusal 99

1. **IMP7D**

**If “EXP7 = 01-02,”** Have you received compensation for these material damages from your insurances?

Yes 01 => IMP7E

No 02 => IMP8

DNK 98 => IMP8

Refusal 99 => IMP8


**IMP7E**

**If “EXP7 = 01-02,”** If you do not mind, could you tell us approximately how much was this compensation in dollars?

Less than $5,000 02

Between $5,001 and $10,000 03

Between $10,001 and $15,000 04

Between $15,001 and $20,000 05

Between $20,001 and $25,000 06

Between $25,001 and $30,000 07

Between $30,001 and $35,000 08

Between $35,001 and $40,000 09

Between $40,001 and $45,000 10

Between $45,001 and $50,000 11

Between $50,001 and $55,000 12

Between $55,001 and $60,000 13

Between $60,001 and $65,000 14

Between $65,001 and $70,000 15

Between $70,001 and $75,000 16

Between $75,001 and $100,000 17

More than $100,000 18

DNK 98

Refusal 99

1. **SAN1**

The last part of the questionnaire addresses various individual characteristics that may influence people’s behavior when they are faced with flooding. Once again, please remember that all your answers will remain strictly confidential. Generally, would you say that your physical health is:

READ OUT THE CHOICES

Excellent 01

Very good 02

Good 03

Fair 04

Poor 05

DNK 98

Refusal 99

1. **SAN2**

Generally, would you say that your mental health is:

READ OUT THE CHOICES

Excellent 01

Very good 02

Good 03

Fair 04

Poor 05

DNK 98

Refusal 99

1. **SAN3**

Thinking about the amount of stress in your life, would you say that most of your days are:

READ OUT THE CHOICES

Not stressful at all 01

Not very stressful 02

Slightly stressful 03

Quite stressful 04

Extremely stressful 05

DNK 98

Refusal 99

1. **SAN4**

Have you ever been diagnosed by a doctor as having chronic health problems affecting your respiratory system?

Yes 01

No 02

DNK 98

Refusal 99

1. **SAN5**

Have you ever been diagnosed by a doctor as having chronic health problems affecting your circulatory system?

Yes 01

No 02

DNK 98

Refusal 99

1. **SAN6**

Have you ever been diagnosed by a doctor as having chronic health problems affecting your nervous system, psychological health or behavior?

Yes 01

No 02

DNK 98

Refusal 99

1. **SAN7**

Have you ever been diagnosed by a doctor as having chronic health problems other than the ones we just spoke of?

Yes 01

No 02 => INC1

DNK 98 => INC1

Refusal 99 => INC1

1. **SAN8**

If “SANT4, SANT5, SANT6, **OR** SANT7 = 01,” what are these chronic health problems, starting with the most important? *Note to the interviewer: maximum of 5 diagnoses.*

Write the diagnosis (or diagnoses) named in order of importance.

1. ____________

2. ____________

3. ____________

4. ____________

5. ____________

DNK 98

Refusal 99

1. **INC1**

Does a physical or mental condition or a health problem reduce the quantity or type of activities that you can do at home, work or school, or others, for example, travel or recreational activities? Would you say:

READ OUT THE CHOICES

Yes, often 01

Yes, sometimes 02

No, never 03

DNK 98

Refusal 99

1. **INC2**

Do you constantly or periodically have trouble hearing (even when using a hearing aid), seeing (even when wearing eyeglasses), communicating (even in your own language), walking, climbing stairs, bending down, reaching or grasping an object, learning or doing other similar activities? Would you say:

READ OUT THE CHOICES

Yes, often 01

Yes, sometimes 02

No, never 03

DNK 98

Refusal 99

1. **SOU1**

In the past 12 months, has someone helped you when needed, for example, to go to the doctor’s, when you were bedridden, to do your housework or prepare your meals if you were unable to do it yourself?

Yes 01 => SOU2

No 02 => AGE1

I haven’t needed any help 03 => AGE1

DNK 98 => AGE1

Refusal 99 => AGE1

1. **SOU2**

If “SOU1 = 01,” among these persons who helped you, how many lived in your neighborhood, but in a different home? By “neighborhood,” we mean anywhere within a 15- to 20-minute walk from your home, when you’re walking at a normal, steady pace.

WRITE THE NUMBER OF PERSONS: _________

DNK 98

Refusal 99

1. **SOU3**

If “SOU1 = 01,” among these persons who helped you, how many lived less than 80 km (50 miles) from your home, but not in the same neighborhood as you?

WRITE THE NUMBER OF PERSONS: _________

DNK 98

Refusal 99

1. **AGE1**

In closing, I will ask you a few demographic and socio-economic questions. They will be used only to classify the answers according to each respondent’s profile. First, how old are you?

WRITE THE AGE HERE: ___________

*Note to the interviewer: if the participant refuses, ask if he/she would accept to say in which age stratus he/she falls.*

READ OUT THE CATEGORIES

18–24 01

25–29 02

30–34 03

35–39 04

40–44 05

45–49 06

50–54 07

55–59 08

60–64 09

65–69 10

70–74 11

75–79 12

80–84 13

85–89 14

90 and over 15

DNK 98

Refusal 99

1. **NAT1**

Were you born:

READ OUT THE CHOICES

In Canada 01 => SCO1

In a country other than Canada 02 => NAT2

DNK 98 => SCO1

Refusal 99 => SCO1

1. **NAT2**

If “NAT1 = 02,” in total, how many years have you lived in Canada, since your first time here?

READ OUT THE CATEGORIES

Less than 1 year 01

From 1 to 4 years 02

From 5 to 9 years 03

10 years or more 04

DNK 98

Refusal 99

1. **SCO1**

Which is the highest education level you have attained?

SPONTANEOUS ANSWER. WRITE the answer

No diploma 01

Elementary school 02

Partial secondary school (Sec I to IV) 03

High school diploma (Sec V or Grade 12) 04

Partial studies in a general and vocational college or trade

school or partial professional training 05

Diploma or certificate from a general and vocational college, a

trade school or professional training 06

Partial university education 07

University degree 08

Other. Please specify 09

DNK 98

Refusal 99

1. **ECO1**

How would you describe your main occupation? Would you say:

READ OUT THE CHOICES

Full-time worker (salaried or self-employed) 01

Part-time worker (salaried or self-employed) 02

Student 03

Retiree or pensioner 04

Off work because of a long-term illness 05

Employment insurance recipient 06

Social assistance recipient 07

Other. Please specify: _______________ 08

DNK 98

Refusal 99

1. **ECO2A**

At what do you estimate your gross annual income (i.e. before deductions) considering all your income sources (e.g. pensions, salaries, scholarships, etc.)? Was it …

READ OUT THE CATEGORIES

Less than $10,000 01

Between $10,000 and $20,000 02

Between $20,001 and $30,000 03

Between $30,001 and $40,000 04

Between $40,001 and $50,000 05

Between $50,001 and $60,000 06

Between $60,001 and $70,000 07

Between $70,001 and $80,000 08

Between $80,001 and $90,000 09

Between $90,001 and $100,000 10

More than $100,000 11

DNK 98

Refusal 99

1. **ECO2B**

If “CM1 ≠ 01,” how many persons, including yourself, have contributed to this income in the last 12 months?

WRITE THE NUMBER OF PERSONS: _________

DNK 98

Refusal 99

**129 :**

**COR1**

If there were a second phase to this study, would you accept to participate?

Yes 01

No 02

**130:**

**COR2**

If “COR1 = 01,” At which e-mail address, landline or cellphone number could we reach you? Once again, please remember that this information will remain strictly confidential and will not be used for any other purpose.

WRITE THE E-MAIL ADDRESS: _________

WRITE THE CELLPHONE NUMBER: _______

WRITE THE LANDLINE NUMBER: _________
